# Life cycle sustainability assessment of a multi-technology action for food loss and waste prevention

**DOI:** 10.12688/openreseurope.22916.1

**Published:** 2026-02-13

**Authors:** María Jesús Muñoz-Torres, Idoya Ferrero-Ferrero, José Vicente Gisbert-Navarro, Elena Escrig-Olmedo

**Affiliations:** 1 Sustainability of Organizations and CSR Management Research Group and IUDESP Research Institute, Universitat Jaume I Faculty of Law and Economics, Castellón de la Plana, Valencian Community, 12071, Spain

**Keywords:** Life Cycle Assessment, Food Loss and Waste Prevention and Reduction action, Technology, Sustainability

## Abstract

**Background:**

Food waste persists as a global challenge, with upstream preventive technological innovations still insufficiently evaluated for their sustainability performance despite policy pressure and the Sustainable Development Goals. The objective of this article is to present a sustainability assessment, from a life-cycle perspective, of an innovative food waste prevention and reduction (FLWPR) action that integrates multiple technologies within the fresh potato supply chain. The intervention applies to a pilot phase technology that consists of advanced imaging and sensor-based detection system to identify internal defects in potatoes early in the supply chain. Potatoes identified as defective are redirected for valorization using commercially available technology into fifth-range products, animal feed, or starch.

**Methods:**

The multi-technological FLWPR action is assessed by applying a Sustainability Life Cycle Assessment using the EF 3.1 method for environmental impacts, a SHDB-based method for social impacts, and Life Cycle Costing for the economic dimension.

**Results:**

Results demonstrate a substantial reduction in food loss and waste, a reduction in the impacts of the three pillars of sustainability and a successful implementation of circular economy practices.

**Conclusions:**

The contribution of this work lies in providing one of the first holistic life-cycle sustainability evaluations of an upstream preventive technological FLWPR action. It demonstrates how the impacts observed during a pilot phase of a technological intervention can be effectively complemented by already commercialized technologies, thereby generating significant system-wide benefits. Moreover, the work highlights pathways to reinforce circularity and enhance sustainability across food supply chains.

## 1. Introduction

Nowadays, the term SDGs (Sustainable Development Goals) has become deeply embedded in public discourse, pretending collective commitment to sustainable development. However, global indicators reveal a stark divergence between rhetoric and reality. According to the latest report by the World Meteorological Organization, the average global surface temperature in 2024 was the highest recorded since systematic measurements began in 1880, and global CO _2_; emissions continued their upward trajectory, reaching a new record in 2024.

From a social perspective, households across all continents wasted the equivalent of more than one billion meals every day in 2022, while 783 million people faced hunger and one third of the global population experienced food insecurity. ^1^ Despite this profound paradox, governments (e.g., Directive (EU) 2025/1892), society, and organizations must continue working toward achieving SDG 12.3 “ *By 2030, halve per capita global food waste at the retail and consumer levels and reduce food losses along production and supply chains, including post-harvest losses*”. In this context, a critical question arises: do we apply the tools and methodologies necessary to effectively manage and control the environmental, social, and economic impacts of our decisions while striving to reduce food waste?

Life Cycle Assessment (LCA) has emerged as a comprehensive methodology for measuring impacts across the entire life-cycle of a product or service. ^3, 4^ Initially developed from an environmental perspective, LCA has progressively expanded to cover economic and social dimensions, giving rise to the concept of Sustainability Life Cycle Assessment (SLCA), which integrates all three pillars of sustainability. ^2^ Despite this evolution, a significant imbalance persists in the maturity of these dimensions. Environmental LCA is well established, while economic (beyond Life Cycle Costing-LCC) and social assessments remain comparatively underdeveloped, making holistic evaluations consistent with ISO standards (ISO 14040/44) ^5, 6^ across all three dimensions particularly challenging.

Within the food waste prevention and reduction topic, climate change has received the greatest attention as an impact category. ^7^ However, there is a pressing need to broaden the scope of assessment to include a wider range of environmental (like water use, eutrophication, ecotoxicity or mineral resource scarcity, among others), economic, and social impacts. In this domain, social LCA, is the least developed and applied. Recent updates to tools such as the food waste prevention calculator, ^8^ promoted by European Commission Joint Research Centre (EC-JRC), have begun to address this gap, offering data information on the nutritional value of the food waste avoided, and adding some social positive messages for users, ^9^ although from a very limited perspective.

Technological approaches to food waste management have been extensively studied, with substantial research arch focusing on downstream treatment strategies such as anaerobic digestion, heat-moisture processing, and composting. While these options help mitigate environmental impacts, they are positioned at the lower tiers of the food waste prevention and reduction hierarchy. This hierarchy, consistent with the Waste Framework Directive, clearly prioritizes prevention measures of food waste along the entire food supply chain before it becomes waste. ^10^ Recent updates of the hierarchy ^11^ further clarify the distinction between “prevention” and “waste treatment,” underscoring the need to shift attention upstream. In contrast, the sustainability assessment of technological actions aimed at preventing food waste has received comparatively limited scholarly attention. ^12^ Given their potential to avoid impacts before they occur, such preventive technological innovations may offer more straightforward and significant contributions to sustainability yet remain insufficiently explored in the current literature.

The objective of this article is to present a sustainability assessment, from a life cycle perspective, of an innovative action for food waste prevention and reduction (FWPR) that integrates multiple technologies. The FLWPR solution under study combines a pilot-scale advanced novel detection system, based on imaging and sensor technologies, to identify defects in potatoes at the platform level before distribution or retail, with existing recycling technologies that valorize potatoes identified as prone to rapid spoilage. This article, published as a pre print in, ^13^ contributes to the literature in three main ways: (i) by conducting a comprehensive sustainability evaluation using widely recognized scientific standards to assess environmental, social, and economic impacts from a life cycle perspective; (ii) by linking technical results from life cycle impact measurement to the management system of the prevention and reduction action, highlighting hotspots, risks and recommendations for improvement; and (iii) by evaluating a set of technologies at different levels of technological maturity, demonstrating how their combination can maximize the effectiveness of food waste prevention and reduction strategies, with a particular focus on the upper tiers of the food waste hierarchy, which remain underrepresented in scientific research.

This article is structured as follows. Following this introduction, Section 2 presents a literature review on environmental, social, and economic impact measurement, with particular emphasis on food waste prevention and reduction. Section 3 describes the methodology for the life cycle assessment of the FLWPR action. Section 4 discusses the results. Section 5 concludes with key insights and recommendations.

## 2. Literature review: Life cycle assessment in food loss and waste prevention, reduction and treatment

LCA plays a central role in the decision-support tools for selecting optimal FLWPR actions, offering a systematic approach to identifying impact hotspots, informing strategic choices, and promoting sustainability-oriented developments in technology, policy, and resource management. ^7^ Despite its potential, few studies have approached LCA as a holistic instrument capable of assessing the environmental, social, and economic sustainability of FLWPR actions.

Environmental LCA has been the most extensively developed and applied approach within the FLWPR field. This focus is also aligned with open access tools such as the Food Waste Prevention Calculator, ^14^ promoted by the European Commission Joint Research Centre (EC-JRC). Recent works, such as Muñoz-Torres et al. ^15^ and Domingo-Morcillo et al., ^16^ have applied environmental LCA to a portfolio of food loss and waste prevention and reduction actions of different types. These assessments follow the ISO 14040/44 standards, ^5, 6^ which define the methodological framework through four iterative phases: (i) goal and scope definition, (ii) life cycle inventory, (iii) life cycle impact assessment, and (iv) interpretation. Because the outcome of an LCA depends on the specific goal, functional unit, parameters, and methodological choices adopted, the results are typically intended for a particular purpose and thus become largely incomparable across studies. Despite this limitation, several works have examined how methodological aspects in LCA design may influence results in this topic ^7^ or have evaluated the suitability of different life cycle impact assessment methods for analyzing the environmental dimension of FLWPR actions. ^16^

In combination with environmental life cycle assessment, some studies have applied life cycle costing to jointly analyze the environmental and economic impacts of food waste management options. These analyses predominantly focus on comparing different FW treatment technologies. For instance, Liu et al. ^17^ evaluated four treatment alternatives (anaerobic digestion, black soldier fly bioconversion, composting, and landfilling), using both LCA and LCC. Their findings indicate that, environmentally, anaerobic digestion performs better than the other alternatives; however, the LCC analysis reveals that it generates the smallest economic return (USD 5.16), while landfilling provides the highest (USD 14.22).

In this context, different LCC approaches have been applied depending on the scope of the economic analysis and the extent to which additional economic implications, such as externalities, are considered. Accordingly, three types of LCC are commonly distinguished: conventional LCC (C-LCC), environmental LCC (E-LCC), and social LCC (S-LCC). Conventional LCC is the most widely applied approach, likely because environmental and social economic parameters are subject to greater uncertainty and more limited data availability. ^18^ As an illustrative example regarding E-LCC, Kim et al. ^19^ assessed the costs of eight food waste disposal options (dry feeding, wet feeding, composting, anaerobic digestion, co-digestion with sewage sludge, food waste disposers, incineration, and landfilling) monetized greenhouse gas emissions. Their findings indicated that landfilling was the least costly option, followed by co-digestion. Regarding S-LCC, Albizzati et al. ^20^ applied a societal life cycle costing approach, focusing on a welfare-economic assessment ^21^ to evaluate five standalone case studies involving wet animal feed, protein-concentrated animal feed, and the production of lactic, polylactic, and succinic acids from food waste. Their results showed that producing animal feed from food waste reduced global warming impacts and socio-economic burdens compared with conventional feed products.

With respect to Social Life Cycle Assessment (S-LCA), it is the least developed and most recent of the three approaches. In this case, the most widely used guidelines are those proposed by the UN Environment Programme in collaboration with the Society of Environmental Toxicology and Chemistry, which provide a framework for conducting S-LCA. ^22– 25^ In this respect, Goldáraz-Salamero et al. ^18^ conducted a comprehensive review of the literature on food loss and waste valorized as feed and found that only 3 out of the 68 studies applied S-LCA. All studies incorporate at least one indicator related to labor welfare, such as working hours or job creation, although none of these three studies clearly specified the type of S-LCA approach adopted. The absence of a clearly defined and comprehensive theoretical framework for social sustainability continues being a major obstacle to the broad implementation and rigorous application of S-LCA, ^26^ underscoring a critical gap and presenting a valuable opportunity for future research to advance and refine this methodology.

Focusing on studies that apply LCA to the assessment of technological solutions, and in line with the discussion presented earlier, as highlighted by Mouat, ^12^ the majority of research addresses solutions classified under food waste treatment [e.g., ^16, 27, 28^]. Specifically, technologies such as composting, incineration, landfilling, anaerobic digestion, hydrothermal carbonization, gasification, pyrolysis, and biochemical methods are primarily examined with regard to their potential for recycling, nutrient recovery, or energy recovery within food waste management systems.

Although there is a consensus in the literature that preventing food loss and waste is generally considered a more sustainable strategy than treatment approaches, ^12, 29^ empirical evidence supporting this assumption remains limited, as many technologies remain insufficiently assessed. ^30^ In fact, some studies reveal that certain technological solutions, such as producing juice from overripe fruits already displayed for sale, may generate positive (unfavorable) net environmental impacts. In these cases, the additional resources used for transportation and electricity outweigh the environmental benefits gained from avoiding food waste. ^15^ Along similar lines, within strategies aimed at extending product shelf-life, Settier-Ramirez et al. ^31^ emphasize that the implementation of activated packaging introduces additional environmental burdens, primarily due to the resource requirements involved in producing and stabilizing the coating. Therefore, a comprehensive sustainability assessment of activated packaging must consider whether the environmental costs incurred during its production are outweighed by the potential benefits of reducing food waste through extended shelf-life.

There is a significant knowledge gap regarding the sustainability assessment of food loss and waste (FLW) prevention technologies. Addressing this gap requires outcome-oriented analyses that move beyond technical performance ^12^ and comprehensively assess their environmental, social, and economic impacts.

## 3. Life cyle analysis method

In this section, this study adopts a life-cycle approach based on the methodological framework established by the ISO 14040/44 standards. ^5, 6^ These phases are applied to the assessment of environmental, social, and economic impacts, thereby ensuring a comprehensive and integrative evaluation of the system. Note that the final phase of the analysis, the life cycle interpretation, is presented in the Results and Discussion section.

This assessment is contextualized in a technological FLWPR action implemented by a leading food retailer, which plays a key role in testing innovative solutions at the upstream level of the food supply chain. This action targeted the fresh potato along the supply chain and aims to reduce food waste by enhancing quality control. The intervention involved the implementation of an innovative technology currently at the pilot stage of development. In particular, an advanced detection system was employed, based on imaging and sensor technologies, to identify defects in potatoes at the platform level before products reached distribution or retail. By enabling the early detection of non-visible defects, the system facilitated more accurate sorting and the redirection of defective potatoes toward alternative uses, rather than discarding them as waste.

Data collection was carried out through a series of meetings and a structured questionnaire within the framework of the ToNoWaste project. The participants in this study are members of the consortium of the ToNoWaste Horizon Europe Project. All participants had previously signed the Grant Agreement and the Consortium Agreement, which included informed consent for their participation in the research.

### 3.1 Goal and scope

**Figure 1.**
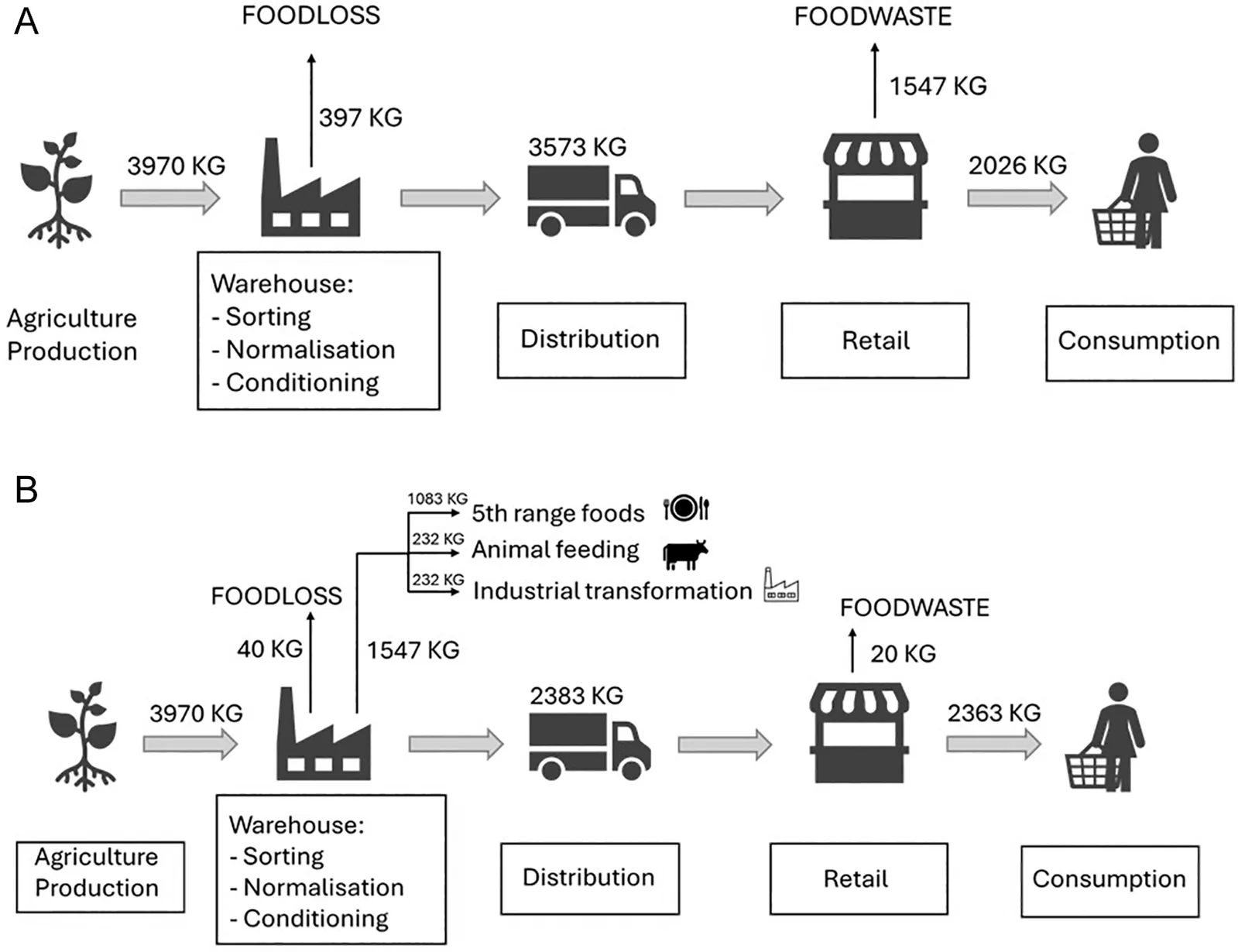
Characterization of scenarios. Comparison of food flows and food waste quantification before ( *ex-ante *) and after the implementation of the food waste prevention and reduction action: (A) *ex-ante* scenario without the reduction action; (B) scenario after the implementation of the technological action. Source: Own elaboration.

The objective of this LCA is to evaluate the sustainability of a technological FLWPR action and to promote circularity within the food system. The declared unit for the assessment is 1 kg of fresh potato. A comparative analysis is conducted between an ex-ante scenario (prior to the implementation of the FLWPR intervention) and an ex-post scenario (following its implementation). Figure 1 presents the system boundaries considered in the assessment for both scenarios. Figure 1 illustrates the flow and quantification of food, food loss, and waste, considering the scope of the pilot phase in which the technology was tested.

In the ex-ante scenario, Figure 1A depicts the waste generated across the different phases. At this stage, the problem identified by the leading food retailer was that, in stores, a percentage of fresh potato bags were rejected due to containing one or more units that did not meet quality standards, thereby posing the risk of this issue reaching consumers. Consequently, the retailer decided to intervene.

In the ex-post scenario, Figure 1B shows the effects of the intervention on food loss and food waste. An advanced detection system equipped with appropriate sensors was installed at the sorting stage of the packaging line to identify and remove defective potatoes with a high risk of causing losses before distribution. This preventive approach was designed to achieve a dual outcome: (i) reducing food loss and waste, and (ii) optimizing the food waste priority hierarchy within the potato supply chain. As a result, the defect detection rate increased substantially, rising from 10% to 40%. Potatoes identified as defective were redirected for valorization using commercially available technology into fifth-range products (ready-to-eat meals), animal feed, or starch, thereby enhancing circularity and contributing to a significant reduction in food waste generation. In conclusion, the food loss and waste reduction resulted in a reduction of 0.47 kg of waste per kilogram of potatoes processed and simultaneously an increase of 0.39 kg valorized per kilogram of potatoes processed.

### 3.2 Life cycle inventory

The data for the characterization and evaluation of the action were provided by the food retailer through four online meetings held July 2024, February 2025, April 2025 and May 2025 with the Head Manager of Innovation and R&D, together with two technicians from the R&D department. Additional data was collected on waste quantification and resource consumption (see ^32^). To provide the life cycle inventory, researchers also accessed Ecoinvent and SHBD databases using the SimaPro license. Note that raw data and extended data is available at. ^32^

### 3.3 Life cycle impact assessment

To estimate a comprehensive range of environmental, social, and economic impact categories integrating the life cycle approach, different methods were applied. From the environmental dimension, this study applies the EF 3.1 method, which is the recommended method by the European Commission (EU) 2021/2279. As highlighted by Domingo-Morcillo et al., ^16^ this method is considered the most suitable since it provides a single score across 16 science-based impact categories, offering sufficient detail to identify critical points and propose corrective actions. From the social dimension, the social impacts are assessed using the SHBD method, which is consistent with the Social Life Cycle Assessment proposed by UNEP. From the economic dimension, a Life Cycle Costing technique is applied.

Additionally, given the specific problem analyzed, this study corrects the impact results by the FLW ratio. In this way, the analysis explicitly considers the inefficiencies attributable to food loss and waste, thereby ensuring that the evaluation accurately reflects the environmental, social, and economic impacts per kilogram of fresh potato.

## 4. Results and discussion

The comparison between the two scenarios was conducted both at the level of individual impact categories and at the aggregated level, using a single score that represents the sum of the weighted impact categories. The next subsections present the results by sustainability dimension.

### 4.1 Environmental impact assessment

**Figure 2.**
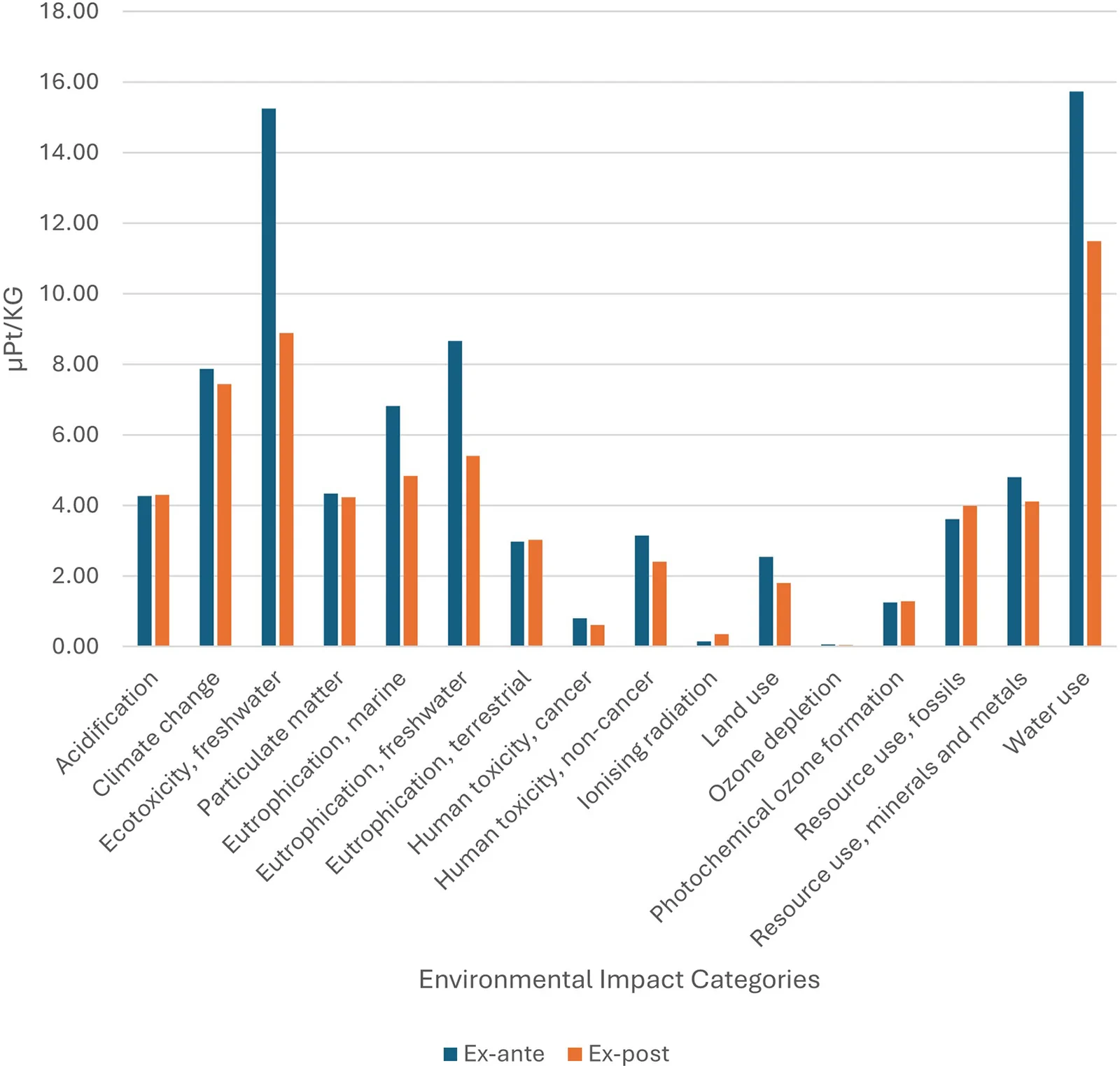
Environmental impact assessment. Comparative assessment of environmental impacts between the ex-ante and ex-post scenario, expressed as weighted results for each Environmental Footprint (EF) 3.1 impact category. Source: own elaboration.

Figure 2 shows the environmental results of the ex-ante and ex-post scenarios. The environmental sustainability assessment of the technological action implemented revealed significant environmental benefits across multiple impact categories. Specifically, the redistribution of 0.47 kg of potatoes yielded a total environmental reduction of 18.02 μPt/kg, with substantial decreases observed in ecotoxicity, water consumption, and eutrophication (see Table 1). The impact category with a higher contribution to the overall environmental impact in both scenarios is “Water Use”. The main source of this impact can be associated with the potato production stage. Accordingly, the improvement proposal should prioritize the development of new measures that specifically address this issue. For the current solution, the margin for further improvement is limited but remains feasible by directly reducing the amount of food wasted. The second most relevant impact category is “Climate Change,” followed by “Acidification” and “Particulate Matter.” Potential improvements in these categories could be achieved through efficiency studies focused on the transformation process, where technological innovations linked to reduced resource use are expected to play a pivotal role

**Table 1.** Environmental impact reduction by kg. of fresh potato.

Impact categories	Impact reduction (μPt/KG)
Acidification	-0.04
Climate change	0.42
Ecotoxicity, freshwater	6.36
Particulate matter	0.10
Eutrophication, marine	1.97
Eutrophication, freshwater	3.25
Eutrophication, terrestrial	-0.05
Human toxicity, cancer	0.20
Human toxicity, non-cancer	0.73
Ionising radiation	-0.20
Land use	0.74
Ozone depletion	0.02
Photochemical ozone formation	-0.02
Resource use, fossils	-0.37
Resource use, minerals and metals	0.69
Water use	4.24
**TOTAL**	**18.02**

Table shows the results of the environmental impact reductions achieved through the implemented food loss and waste prevention and reduction action. Note that a negative value indicates an increase in environmental impact and therefore does not represent a reduction.

Source: own elaboration.

.

Table 1 presents the difference in weighted environmental impacts between the ex-ante scenario (prior to the implementation of the action) and the ex-post scenario (after its implementation). This comparison enables a clear quantification of the changes attributable to the intervention and supports informed decision-making based on the action’s sustainability performance. Overall, Table 1 shows a favorable reduction in total impact and across a large number of impact categories. However, five associated impact categories display an unfavorable evolution after the implementation of the FLWPR action. These categories can be identified as potential environmental risk points, as they indicate an increase in environmental burdens. This outcome is explained by the fact that the saved impacts due to the food waste reduction are not enough to offset the resource consumption and associated impacts of the technologies employed, both for early detection and for valorization processes. Nevertheless, in relative terms, the negative contribution of these 5 categories remains low compared with the overall reduction in impact.

### 4.2 Social impact assessment

The action also resulted in a reduction in social impacts, quantified at 16.62 Pt/kg. In addition, it generated a well-balanced improvement across all five major social impact categories (see Table 2).

**Table 2.** Social impact reduction by kg. of fresh potato.

Impact categories and subcategories	Impact reduction (Pt/KG)
**Labor Rights & Decent Work**	**8.32**
Child Labor	1.06
Forced Labor	0.54
Excessive Working Time	0.53
Wage Assessment	2.66
Poverty	0.86
Migrant Labor	0.35
Collective Bargaining	1.86
Social Benefits	0.45
**Health & Safety**	**2.55**
Injuries and Fatalities	0.84
Toxics and Hazards	1.71
**Human Rights**	**1.94**
Indigenous Rights	0.45
Gender Equity	0.52
High Conflict	0.97
**Governance**	**2.07**
Legal System	0.92
Corruption	1.15
**Community Infrastructure**	**1.74**
Drinking Water	0.48
Improved Sanitation	0.57
Hospital Beds	0.69
**TOTAL**	**16.62**

Table shows the results of the social impact reductions achieved through the implemented food loss and waste prevention and reduction action.

Source: own elaboration.

**Figure 3.**
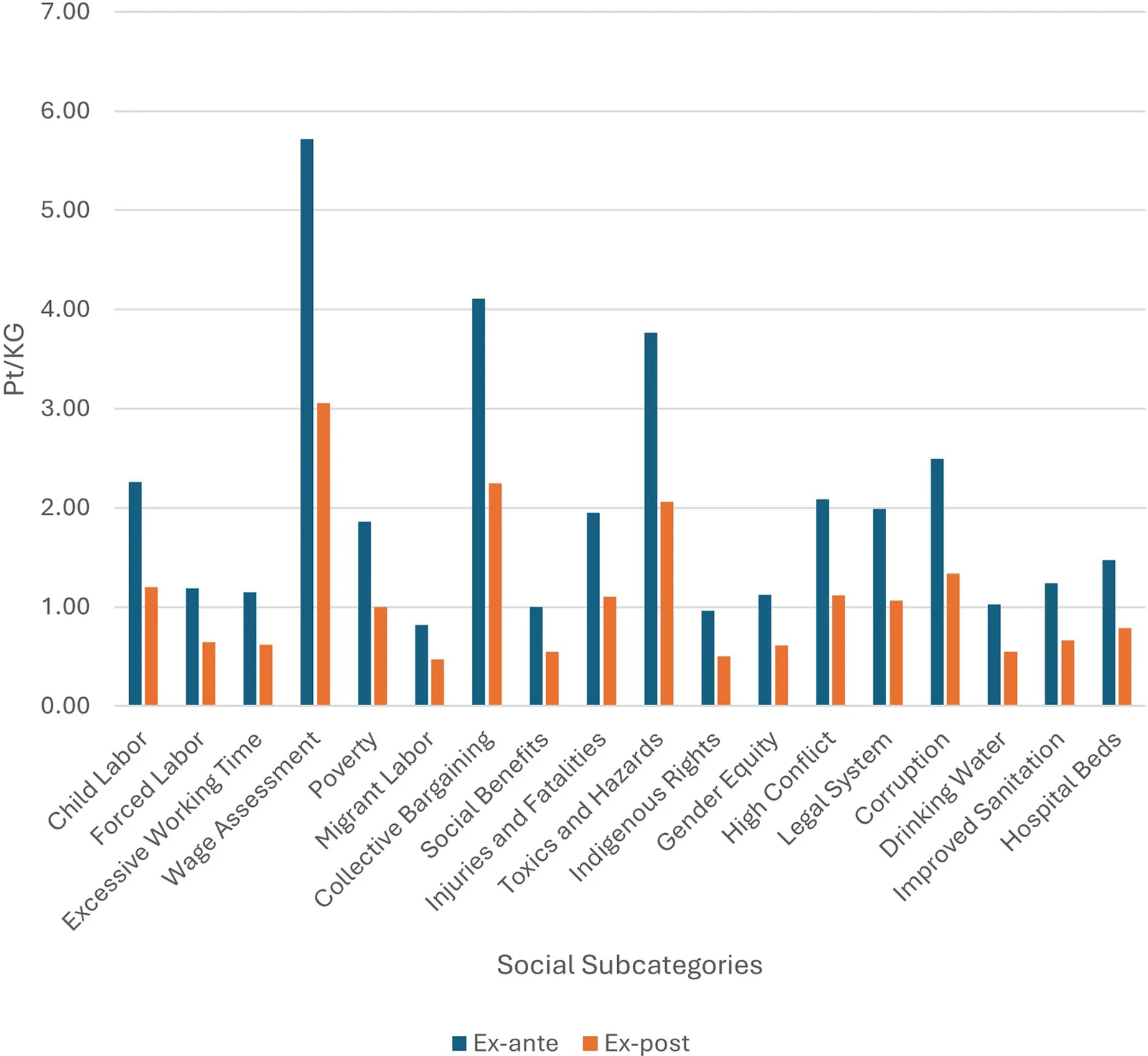
Social impact assessment. Comparative assessment of social impacts between the ex-ante and ex-post scenario, expressed as results for each SHDB impact subcategory. Source: own elaboration.

Figure 3 displays the social results of both scenarios. The social subcategory with the highest contribution corresponds to “Wage Assessment”, with the main contribution originated from labor conditions in the potato production phase. Improvement efforts should therefore prioritize the development of new measures targeting this aspect. The second most relevant impact subcategory is Injuries and Fatalities. In third place, in terms of relative importance, is “Toxics and Hazards”, followed by “Collective Bargaining”. Across all social impact categories, the primary contribution stems from food-related processes. Consequently, improvement strategies should emphasize actions that directly address this source of impact. Regarding the current FLWPR action, the potential margin for improvement is limited but still possible, particularly by further reducing the amount of food loss and waste.

Table 2 presents the difference in social impacts between both scenarios. Table 2 presents the differences in social impacts between the two scenarios. The results indicate that all social impact categories has a reduction. The most substantial decrease is observed in “ *Labor Rights and Decent Work”*, which accounts for a total reduction of 8.32 Pt/kg. Within this category, the largest contributions to the improvement arise from wage assessment (2.66 Pt), collective bargaining (1.86 Pt), and child labour (1.06 Pt). These results highlight that the technological FLWPR action itself generates only a very limited social impact. Consequently, the reductions achieved in all social impact categories can be clearly attributed to the decrease in food loss and waste.

### 4.3 Economic impact assessment

From an economic perspective, the evaluation focuses exclusively on cost. Prior to the implementation of the action, the cost was 2.43 €/kg, largely due to the inefficiencies associated with food loss and waste, which reached rates of 49%. The technological FLWPR action reduced food loss and waste to an optimal level of approximately 2%. This improved resource use, enabled by early food waste and loss detection and the various waste valorization options, allowed for a more efficient allocation of resources. Even considering the additional resources required by the technological solution, the cost per kilogram decreased to 2.29 €/kg, representing a reduction due to the action of 0.14 €/kg. This improvement is explained by an increased efficiency in food handling, enhanced product preservation, and the optimization of human and energy resources. The reduction of waste at the distribution stage not only avoids direct losses but also enhances overall supply chain performance.

## 5. Conclusions

This study presents a comprehensive sustainability assessment of a technological FLWPR action, which integrates multiple technologies to enhance food waste prevention and contribute to the development of a more sustainable food system. The technologies applied operate at different stages of maturity. On the one hand, a pilot-phase technology was tested, designed to prevent waste through the implementation of an advanced detection system. This system employs appropriate sensors installed at the sorting stage of the packaging line to identify and remove potatoes with a high risk of generating losses due to defects, thereby preventing waste before it occurs. On the other hand, market-ready technologies were applied to valorize defective potatoes through the production of fifth-range products (ready-to-eat meals), animal feed, and industrial transformation. The sustainability assessment was conducted using a life cycle approach, integrating the three dimensions of sustainability, environmental, social, and economic, through methods widely recognized in academic research. The system boundaries for this multi-technological solution extended from production to consumption.

The first results based on the FLW rate demonstrated that the FLWPR solution effectively improved the identification of defective potatoes, significantly reducing waste generation and contributing to circular economy practices by redirecting defective potatoes to alternative uses. The sustainability results revealed positive impacts across all three dimensions. Environmentally, the intervention achieved notable reductions in ecotoxicity and water use, reflecting improved resource efficiency. Socially, the pilot contributed to enhanced labor conditions, with measurable progress in wage assessment, collective bargaining, and the reduction of risks related to forced and child labor. Economically, the intervention proved cost-effective by lowering waste-related losses and improving supply chain efficiency. A deeper analysis of the results highlights critical areas requiring continued attention, particularly certain environmental impact categories that showed increased risk.

This article makes several contributions to the field of sustainability assessment of technological FLWPR actions. First, it provides a comprehensive sustainability evaluation that integrates environmental, social, and economic dimensions from a life cycle perspective, applying widely recognized scientific standards. This is particularly relevant in the technological assessment domain, where there is a notable lack of studies that include social impact evaluation. The methodology presented here offers a framework that can be applied not only to determine the economic feasibility of scaling up technologies but also to assess their contribution to sustainable development.

Second, the article establishes a direct link between the technical results obtained from life cycle impact measurement and the management system of the prevention and reduction action. This connection allows for the identification of hotspots, risks, and recommendations for improvement. From a practical standpoint, it is not only essential to decide whether a technology should be adopted but also to understand how it can be continuously improved within a framework of ongoing optimization.

Third, the study evaluates a set of technologies at different levels of maturity, demonstrating how their combination can maximize the effectiveness of food waste prevention and reduction strategies. Particular emphasis is placed on the upper tiers of the food waste hierarchy, which remains underrepresented in scientific research, as most existing studies focus primarily on waste treatment rather than prevention. The positive results of this assessment suggest that this multi-technological approach may be of interest to other companies involved in potato distribution and sales. By adopting similar strategies, these companies could directly contribute to Sustainable Development Goal 12.3 as well as to other environmental, social, and economic goals associated with broader impact categories.

This paper acknowledges several limitations related to empirical analysis. One of the main limitations concerns the social datasets available in the specialized databases associated with the food analyzed, which are based on general averages without the possibility of selecting a dataset specific to the variety and region studied. As social databases continue to expand and improve, future evaluations will be able to achieve greater accuracy. Another limitation arises in the economic dimension, where the assessment currently considers only cost as an impact category. Future research should explore additional categories such as profit margins, eco-efficiency, and other indicators that can provide a more comprehensive understanding of economic performance. A further limitation is linked to the pilot phase of the early detection technology applied to potatoes. Previous studies suggest that the performance of such technologies may vary when scaled to market-level applications. If this methodology eventually reaches commercial maturity, repeating the sustainability assessment would allow for a comparative analysis to identify potential deviations across different stages of technological development.

Overall, this study confirms the value of integrating technological innovation with sustainability assessment frameworks to prevent food loss and waste. The findings support the scaling up of such solutions within the retail sector and provide actionable insights for stakeholders seeking to enhance sustainability performance across food supply chains.

## Ethical approval statement

This study involved human participants. Specifically, the owners of the action provided information regarding the technological FLWPR action within the ToNoWaste project through meetings and a questionnaire. The research was conducted in accordance with the ethical guidelines and regulations of the ToNoWaste Horizon Europe project and its coordinating institution. The study protocol was reviewed and approved by the Human Research Ethics Committee of Universitat Jaume I (Spain), with the ethical approval file number CEISH/56/2023.

## Consent to participate

The participants in this study are part of the consortium of the ToNoWaste Horizon Europe Project and they signed the Grant Agreement and the Consortium Agreement (25/05/2022), which included a written informed consent to participation in research.

## Declaration of generative AI and AI-assisted technologies in the manuscript preparation process

During the preparation of this work the authors used Microsoft Copilot tool (Microsoft, 2025) in order to improve readability and language. After using this tool, the authors reviewed and edited the content as needed and take full responsibility for the content of the published article.

## Data Availability

Repositori UJI, TONOWASTE (Towards a New Zero Food Waste Mindset Based on Holistic Assessment). https://repositori.uji.es/handle/10234/775844. ^32^ This project contains the following underlying data:
• Dataset of environmental, social and economic impact: a Multi-Technology Action for Food Loss and Waste Prevention. Data is available under the terms of the CC-BY 4.0 license. http://creativecommons.org/licenses/by/4.0/
