# Life cycle sustainability assessment of a multi-technology action for food loss and waste prevention

**DOI:** 10.12688/openreseurope.22916.3

**Published:** 2026-07-09

**Authors:** María Jesús Muñoz-Torres, Idoya Ferrero-Ferrero, José Vicente Gisbert-Navarro, Elena Escrig-Olmedo

**Affiliations:** 1 Sustainability of Organizations and CSR Management Research Group and IUDESP Research Institute, Universitat Jaume I Faculty of Law and Economics, Castellón de la Plana, Valencian Community, 12071, Spain

**Keywords:** Life Cycle Assessment, Food Loss and Waste Prevention and Reduction action, Technology, Sustainability

## Abstract

**Background:**

Food waste persists as a global challenge, with upstream preventive technological innovations still insufficiently evaluated for their sustainability performance despite policy pressure and the Sustainable Development Goals. The objective of this article is to present a sustainability assessment, from a life-cycle perspective, of an innovative food waste prevention and reduction (FLWPR) action that integrates multiple technologies within the fresh potato supply chain and to examine to what extent the food waste hierarchy aligns with multidimensional sustainability criteria. The intervention applies to a pilot phase technology that consists of advanced imaging and sensor-based detection system to identify internal defects in potatoes early in the supply chain. Potatoes identified as defective are redirected for valorization using commercially available technology into fifth-range products, animal feed, or starch.

**Methods:**

The multi-technological FLWPR action is assessed by applying a Sustainability Life Cycle Assessment using the EF 3.1 method for environmental impacts, a SHDB-based method for social impacts, and Life Cycle Costing for the economic dimension.

**Results:**

Demonstrate a substantial reduction in food loss and waste, a reduction in the impacts of the three pillars of sustainability and a successful implementation of circular economy practices.

**Conclusions:**

The contribution of this work lies in providing one of the first holistic life-cycle sustainability evaluations of an upstream preventive technological FLWPR action. It demonstrates how the impacts observed during a pilot phase of a technological intervention can be effectively complemented by already commercialized technologies, thereby generating significant system-wide benefits. Moreover, the work highlights pathways to reinforce circularity and enhance sustainability across food supply chains.

## 1. Introduction

Life Cycle Assessment (LCA) has emerged as a comprehensive methodology for measuring impacts across the entire life cycle of a product or service. ^1, 2^ Initially developed from an environmental perspective, LCA has progressively expanded to cover economic and social dimensions, giving rise to the concept of Sustainability Life Cycle Assessment (SLCA), which integrates all three pillars of sustainability. ^3^ Despite this evolution, a significant imbalance persists in the maturity of these dimensions. Environmental LCA is well established, while economic (beyond Life Cycle Costing-LCC) and social assessments remain comparatively underdeveloped, ^4^ making holistic evaluations consistent with ISO standards (ISO 14040/44) ^5, 6^ across all three dimensions particularly challenging.

Within the food waste prevention and reduction topic, climate change has received the greatest attention as an impact category. ^7^ However, there is a pressing need to broaden the scope of assessment to include a wider range of environmental (like water use, eutrophication, ecotoxicity or mineral resource scarcity, among others), economic, and social impacts. In this domain, social LCA, is the least developed and applied. Recent updates to tools such as the food waste prevention calculator, ^8^ promoted by European Commission Joint Research Centre (EC-JRC), have begun to address this gap, offering data information on the nutritional value of the food waste avoided, and adding some social positive messages for users, ^9^ although from a very limited perspective.

Technological approaches to food waste management have been extensively studied, with substantial research arch focusing on downstream treatment strategies such as anaerobic digestion, heat-moisture processing, and composting. ^4^ While these options help mitigate environmental impacts, they are positioned at the lower tiers of the food waste prevention and reduction hierarchy. This hierarchy, consistent with the Waste Framework Directive, clearly prioritizes prevention measures of food waste along the entire food supply chain before it becomes waste. ^10^ Recent updates of the hierarchy ^11^ further clarify the distinction between “prevention” and “waste treatment,” underscoring the need to shift attention upstream. In contrast, the sustainability assessment of technological actions aimed at preventing food waste has received comparatively limited scholarly attention. ^12^ Given their potential to avoid impacts before they occur, such preventive technological innovations may offer more straightforward and significant contributions to sustainability yet remain insufficiently explored in the current literature. ^12^^,^^13^ Moreover, despite the strategic importance of this hierarchy, few studies have rigorously analyzed whether its prioritization consistently aligns with comprehensive sustainability outcomes. Existing research has predominantly focused on the environmental dimension through LCA (e.g., Refs. 4, 14, and 15), often overlooking the integrated economic and social implications of moving up the hierarchy.

The objective of this article is to present a sustainability assessment, from a life cycle perspective, of an innovative action for food waste prevention and reduction (FWPR) that integrates multiple technologies and to examine to what extent the food waste hierarchy aligns with multidimensional sustainability criteria. The FLWPR solution under study combines a pilot-scale advanced novel detection system, based on imaging and sensor technologies, to identify defects in potatoes at the platform level before distribution or retail, with existing recycling technologies that valorize potatoes identified as prone to rapid spoilage. This article, published as a preprint in, ^16^ contributes to the literature in three main ways: (i) by conducting a comprehensive sustainability evaluation using widely recognized scientific standards to assess environmental, social, and economic impacts from a life cycle perspective; (ii) by linking technical results from life cycle impact measurement to the management system of the prevention and reduction action, highlighting hotspots, risks and recommendations for improvement; and (iii) by evaluating a set of technologies at different levels of technological maturity, demonstrating how their combination can maximize the effectiveness of food waste prevention and reduction strategies, with a particular focus on the upper tiers of the food waste hierarchy, which remain underrepresented in scientific research.

This article is structured as follows. Following this introduction, Section 2 presents a literature review on environmental, social, and economic impact measurement, with particular emphasis on food waste prevention and reduction. Section 3 describes the methodology for the life cycle assessment of the FLWPR action. Section 4 discusses the results. Section 5 concludes with key insights and recommendations.

## 2. Literature review: Life cycle assessment in food loss and waste prevention, reduction and treatment

LCA plays a central role in the decision-support tools for selecting optimal FLWPR actions, offering a systematic approach to identifying impact hotspots, informing strategic choices, and promoting sustainability-oriented developments in technology, policy, and resource management. ^7^ Despite its potential, few studies have approached LCA as a holistic instrument capable of assessing the environmental, social, and economic sustainability of FLWPR actions.

Environmental LCA has been the most extensively developed and applied approach within the FLWPR field. This focus is also aligned with open access tools such as the Food Waste Prevention Calculator, ^17^ promoted by the European Commission Joint Research Centre (EC-JRC). Recent works, such as Muñoz-Torres et al. ^14^ and Domingo-Morcillo et al., ^18^ have applied environmental LCA to a portfolio of food loss and waste prevention and reduction actions of different types. These assessments follow the ISO 14040/44 standards, ^5, 6^ which define the methodological framework through four iterative phases: (i) goal and scope definition, (ii) life cycle inventory, (iii) life cycle impact assessment, and (iv) interpretation. Because the outcome of an LCA depends on the specific goal, functional unit, parameters, and methodological choices adopted, the results are typically intended for a particular purpose and thus become largely incomparable across studies. Despite this limitation, several works have examined how methodological aspects in LCA design may influence results in this topic ^7^ or have evaluated the suitability of different life cycle impact assessment methods for analyzing the environmental dimension of FLWPR actions. ^18^

In combination with life cycle assessment, some studies have applied life cycle costing to jointly analyze the environmental and economic impacts of food waste management options. These analyses predominantly focus on comparing different FW treatment technologies. For instance, Liu et al. ^19^ evaluated four treatment alternatives (anaerobic digestion, black soldier fly bioconversion, composting, and landfilling), using both LCA and LCC. Their findings indicate that, environmentally, anaerobic digestion performs better than the other alternatives; however, the LCC analysis reveals that it generates the smallest economic return (USD 5.16), while landfilling provides the highest (USD 14.22).

In this context, different LCC approaches have been applied depending on the scope of the economic analysis and the extent to which additional economic implications, such as externalities, are considered. Accordingly, three types of LCC are commonly distinguished: conventional LCC (C-LCC), environmental LCC (E-LCC), and social LCC (S-LCC). Conventional LCC is the most widely applied approach, likely because environmental and social economic parameters are subject to greater uncertainty and more limited data availability. ^20^ As an illustrative example regarding E-LCC, Kim et al. ^21^ assessed the costs of eight food waste disposal options (dry feeding, wet feeding, composting, anaerobic digestion, co-digestion with sewage sludge, food waste disposers, incineration, and landfilling) monetized greenhouse gas emissions. Their findings indicated that landfilling was the least costly option, followed by co-digestion. Regarding S-LCC, Albizzati et al. ^22^ applied a societal life cycle costing approach, focusing on a welfare-economic assessment ^23^ to evaluate five standalone case studies involving wet animal feed, protein-concentrated animal feed, and the production of lactic, polylactic, and succinic acids from food waste. Their results showed that producing animal feed from food waste reduced global warming impacts and socio-economic burdens compared with conventional feed products.

With respect to Social Life Cycle Assessment (S-LCA), it is the least developed and most recent of the three approaches. In this case, the most widely used guidelines are those proposed by the UN Environment Programme in collaboration with the Society of Environmental Toxicology and Chemistry, which provide a framework for conducting S-LCA. ^24– 27^ In this respect, Goldáraz-Salamero et al. ^20^ conducted a comprehensive review of the literature on food loss and waste valorized as feed and found that only 3 out of the 68 studies applied S-LCA. All studies incorporate at least one indicator related to labor welfare, such as working hours or job creation, although none of these three studies clearly specified the type of S-LCA approach adopted. The absence of a clearly defined and comprehensive theoretical framework for social sustainability continues being a major obstacle to the broad implementation and rigorous application of S-LCA, ^28^ underscoring a critical gap and presenting a valuable opportunity for future research to advance and refine this methodology.

Focusing on studies that apply LCA to the assessment of technological solutions, and in line with the discussion presented earlier, as highlighted by Mouat, ^12^ the majority of research addresses solutions classified under food waste treatment [e.g., Refs. 18, 29 and 30]. Specifically, technologies such as composting, incineration, landfilling, anaerobic digestion, hydrothermal carbonization, gasification, pyrolysis, and biochemical methods are primarily examined regarding their potential for recycling, nutrient recovery, or energy recovery within food waste management systems.

Although there is a consensus in the literature that preventing food loss and waste is generally considered a more sustainable strategy than treatment approaches, ^12, 31^ empirical evidence supporting this assumption remains limited, as many technologies remain insufficiently assessed. ^32^ In fact, some studies reveal that certain technological solutions, such as producing juice from overripe fruits already displayed for sale, may generate positive (unfavorable) net environmental impacts. In these cases, the additional resources used for transportation and electricity outweigh the environmental benefits gained from avoiding food waste. ^16^ Along similar lines, within strategies aimed at extending product shelf-life, Settier-Ramirez et al. ^33^ emphasize that the implementation of activated packaging introduces additional environmental burdens, primarily due to the resource requirements involved in producing and stabilizing the coating. Therefore, a comprehensive sustainability assessment of activated packaging must consider whether the environmental costs incurred during its production are outweighed by the potential benefits of reducing food waste through extended shelf-life.

There is a significant knowledge gap regarding the sustainability assessment of food loss and waste (FLW) prevention technologies. Addressing this gap requires outcome-oriented analyses that move beyond technical performance ^12^ and comprehensively assess their environmental, social, and economic impacts.

## 3. Life cycle analysis method

In this section, this study adopts a life-cycle approach based on the methodological framework established by the ISO 14040/44 standards. ^5, 6^ These phases are applied to the assessment of environmental, social, and economic impacts, thereby ensuring a comprehensive and integrative evaluation of the system. Note that the final phase of the analysis, the life cycle interpretation, is presented in the Results and Discussion section.

This assessment is contextualized in a technological FLWPR action implemented by a leading food retailer, which plays a key role in testing innovative solutions at the upstream level of the food supply chain. This action targeted the fresh potato along the supply chain and aims to reduce food waste by enhancing quality control. The intervention involved the implementation of an innovative technology currently at the pilot stage of development. In particular, an advanced detection system was employed, based on imaging and sensor technologies, to identify defects in potatoes at the platform level before products reached distribution or retail. By enabling the early detection of non-visible defects, the system facilitated more accurate sorting and the redirection of defective potatoes toward alternative uses, rather than discarding them as waste.

Data collection was carried out through a series of meetings and a structured questionnaire within the framework of the ToNoWaste project. The participants in this study are members of the consortium of the ToNoWaste Horizon Europe Project. All participants had previously signed the Grant Agreement and the Consortium Agreement, which included informed consent for their participation in the research.

### 3.1 Goal and scope

The objective of this LCA is to evaluate the sustainability of a technological FLWPR action and to promote circularity within the food system. The declared unit for the assessment is 1 kg of fresh maincrop potato. The potato is the main non-cereal crop in the EU, with a production of 47.5 million tons in 2023. ^34^ Furthermore, potatoes alone account for 10% of all food waste generated in the EU, placing them as one of the individual products with the greatest impact on waste streams due to their massive production volume and the accumulated losses in both household storage and industrial processing. ^35^

**Figure 1.**
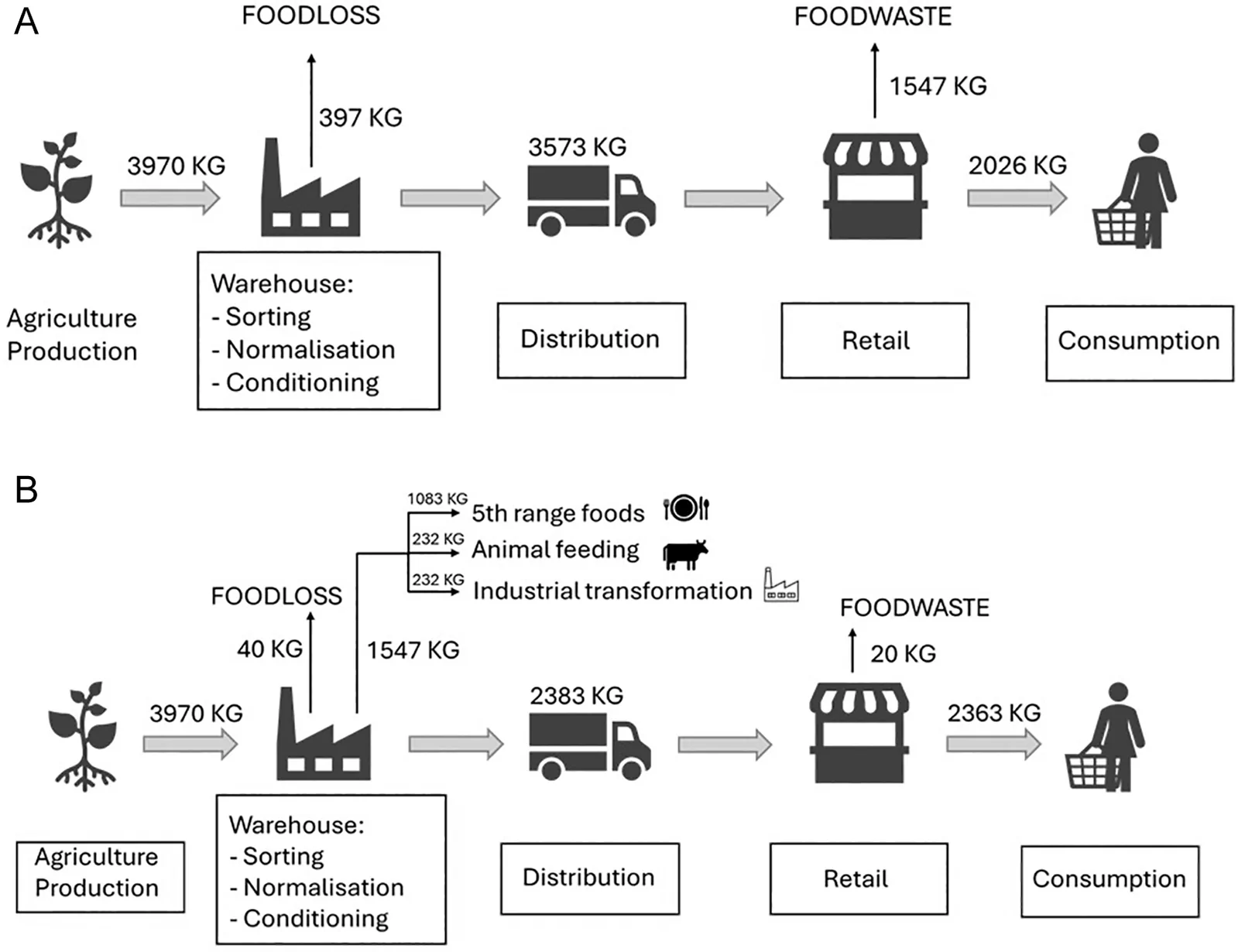
Characterization of accounting-based scenarios. Comparison of food flows and food waste quantification before and after the implementation of the food waste prevention and reduction action: (A) accounting-based scenario without the reduction action; (B) accounting-based scenario after the implementation of the technological action that combines an Advanced detection System to identify and remove defective potatoes with available Technology for optimizing the food loss with fifth-range products, animal feed and starch production. Source: Own elaboration.

This FLWPR action is modeled as a global, site-independent intervention rather than an activity tied to the specific peculiarities of a single region. A comparative analysis is conducted between the accounting-based scenario without and with FLWPR action. To gather the data, the execution period of the FLWPR action was defined from December 1st to December 31st, 2024. However, the duration of effects is extended to encompass the full commercialization period of the potatoes. Figure 1 presents the system boundaries considered in the assessment for both scenarios. This study adopts a cradle-to-gate perspective, extended to include the distribution and retail phases. The system boundary encompasses raw material extraction, manufacturing, distribution and retail. The consumption phase was excluded from the scope as they do not vary between the scenarios considered. Note that food loss and waste generated during the processes included in the system boundary are considered. As the action owner highlighted, the generated food loss and waste is treated through composting processes. Fixed capital investments and infrastructure were excluded from the system boundary as they represent negligible impacts when normalized per declared unit. This exclusion is consistent with standard cut-off criteria, as the contribution of capital goods to the total life cycle impact falls below the established significance threshold.

Figure 1 illustrates the flow and quantification of food, food loss, and waste, considering the scope of the pilot phase in which the technology was tested.

In the accounting-based scenario previous the implementation of the FLPWR action, Figure 1A depicts the waste generated across the different phases. At this stage, the problem identified by the leading food retailer was that, in stores, a percentage of fresh potato bags were rejected due to containing one or more units that did not meet quality standards, thereby posing the risk of this issue reaching consumers. Consequently, the retailer decided to intervene.

In the accounting-based scenario after the implementation of the FLPWR action, Figure 1B shows the effects of the intervention on food loss and food waste. An advanced detection system equipped with appropriate sensors was installed at the sorting stage of the packaging line to identify and remove defective potatoes with a high risk of causing losses before distribution. This preventive approach was designed to achieve a dual outcome: (i) reducing food loss and waste, and (ii) optimizing the food waste priority hierarchy within the potato supply chain. As a result, the defect detection rate increased substantially, rising from 10% to 40%. Potatoes identified as defective were redirected for valorization using commercially available technology into fifth-range products (ready-to-eat meals), animal feed, or starch, thereby enhancing circularity and contributing to a significant reduction in food waste generation. In conclusion, the food loss and waste reduction resulted in a reduction of 0.47 kg of waste per kilogram of potatoes processed and simultaneously an increase of 0.39 kg valorized per kilogram of potatoes processed.

**Figure 2.**
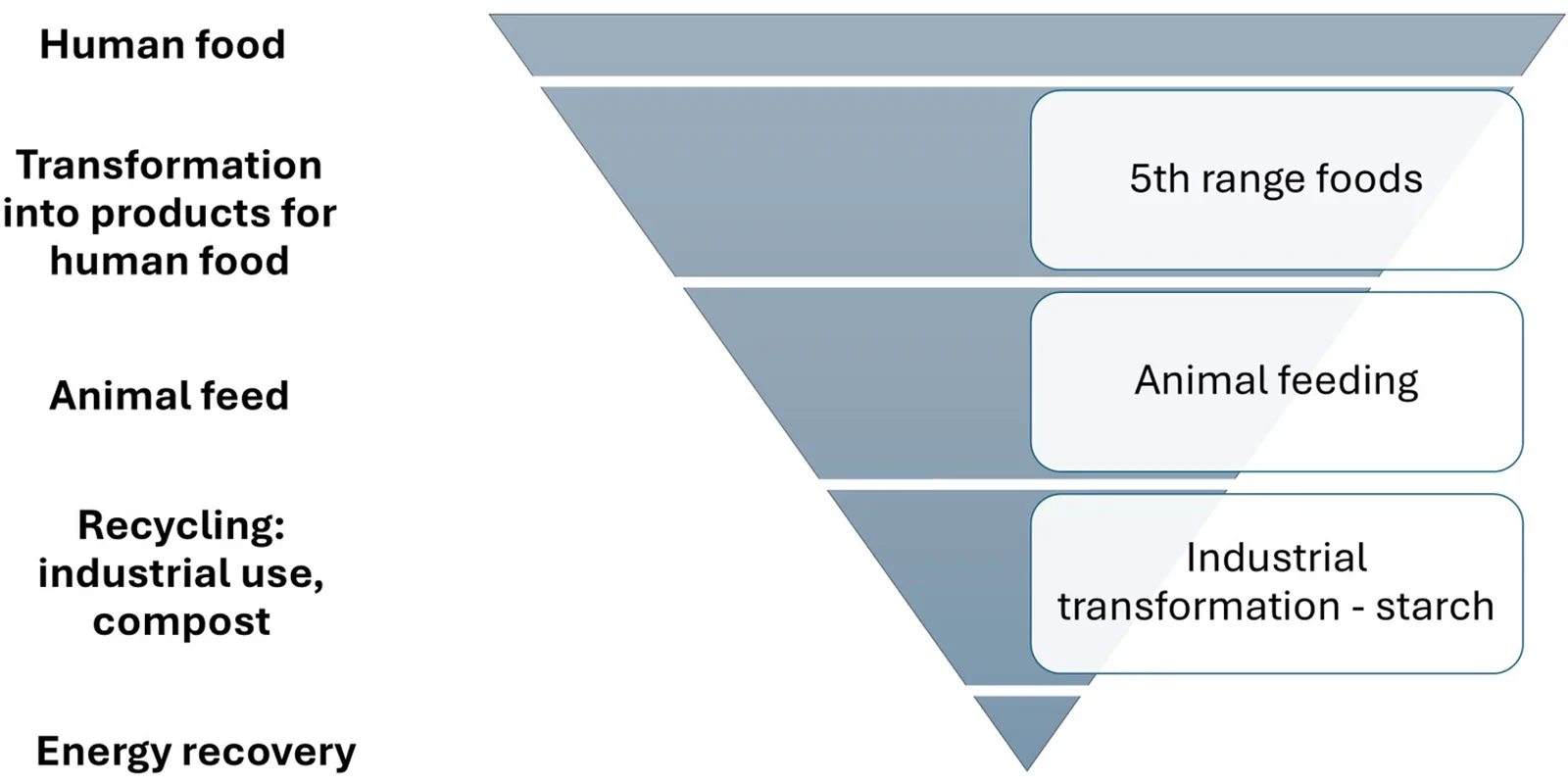
Food waste prevention and reduction hierarchy and strategies developed in the case study. It shows the three food loss reduction strategies implemented in the warehouse and the respective levels related to the food waste prevention and reduction hierarchy. Source: Own elaboration.

Additionally, the study analyzes the sensitivity of sustainability impacts across six new potential scenarios. These scenarios involve varying the distribution of warehouse-generated food loss among the reduction strategies already implemented by the action owner, which are situated at various levels of the food waste hierarchy (see Figure 2). This approach allows us to examine how consistent sustainability performance is of the three FLWPR strategies when compared against the food waste reduction hierarchy. These three strategies are: 5 ^th^ range foods which belongs to transformation into products for human food level, animal feed and starch which is considered as a recycling FWLPR strategy.

The sensitivity analysis assesses 7 scenarios as detailed in Table 1. Scenario A reflects current commercial and industrial conditions, which has been previously presented in Figure 1B as the accounting-based baseline with the FLWPR action. Scenario B aligns with the waste hierarchy by prioritizing the human food recovery strategy, while Scenario C explores the impact of an inverted hierarchy. Scenario D provides a neutral benchmark by distributing loss equally across all strategies. Lastly, Scenarios E1 (5th range foods), E2 (animal feed), and E3 (starch) serve as extreme boundary scenarios to assess the impact of opting for a single recovery pathway.

**Table 1.** Environmental impact reduction by kg of fresh potato.

FLWPR Strategy	Scenario A	Scenario B	Scenario C	Scenario D	Scenario E1	Scenario E2	Scenario E3
5 ^th^ range foods	69.80%	85.00%	5.00%	33.33%	100.00%	0.00%	0.00%
Animal feed	15.14%	10.00%	10.00%	33.33%	0.00%	100.00%	0.00%
Starch	15.14%	5.00%	85.00%	33.33%	0.00%	0.00%	100.00%

### 3.2 Life cycle inventory

The data for the characterization and evaluation of the action were provided by the food retailer through four online meetings held July 2024, February 2025, April 2025 and May 2025 with the Head Manager of Innovation and R&D, together with two technicians from the R&D department. Additional data was collected on waste quantification and resource consumption (see Ref. 36). Table 2 shows the data considered to assess the FLWPR action. All inputs were cross-checked and supervised by an expert in the agri-food sector, who validated their consistency based on extensive industry experience. Based on the collected data, the life cycle inventory was performed in SimaPro, supported by Ecoinvent and the SHBD databases. The EF 3.1 impact assessment method was then applied following, ^14^ jointly with the SHBD methodology. The resulting impact calculations for the three dimensions are provided in the supplementary file. ^36^

**Table 2.** Data gathering to the FLWPR action.

Food Loss and Waste Quantification	Results	Cost
Accounting-based scenario without FLWPR	0.49 kg loss and waste/kg processed	1.24 €/kg potato
	0.00 kg transformed/kg processed	
Accounting-based scenario with FLWPR	0.02 kg loss and waste/kg processed	1.24 €/kg potato
	0.272 kg 5 ^th^ range/kg processed 0.059 kg animal feeding/kg processed 0.059 kg industrial transformation/kg processed	0.1369 €/kg (5 ^th^ range) 0.0561 €/kg (animal feeding) 0.1587€ /kg (industrial transformation)

Resources consumed by the FLWPR action	Data	Cost
Electricity consumption of two sensors and a PC	0.00169 Kwh/kg processed	0.25 €/kwh
Labor requirements associated with two technicians and one supervisor	0.02116 h technician/kg processed	21 €/h
	0.01587 h supervisor/kg processed	35 €/h

To provide the life cycle inventory, researchers also accessed Ecoinvent and SHBD databases using the SimaPro license. Given the relevance of the potato dataset for the LCA, this study highlights that it is sourced from Nemecek and Kägi’s Life Cycle Inventories of Agricultural Production Systems, a widely recognized and robust database in agricultural LCA. The data refer to the ‘Rest of the World’ geography and cover the period 2001–2025. The dataset reports a 100% representativeness, indicating full applicability to the modeled system. Note that raw data and extended data is available at. ^36^

### 3.3 Life cycle impact assessment

To estimate a comprehensive range of environmental, social, and economic impact categories integrating the life cycle approach, different methods were applied. From the environmental dimension, this study applies the EF 3.1 method, which is the recommended method by the European Commission (EU) 2021/2279. The EF 3.1 method applies its specific characterization factors (CFs) to convert elementary flows into 16 impact categories (each one with its own unit). Following the EF 3.1 method, this study has calculated the impact categories normalized and weighted. Weighted impact results were calculated by normalizing characterized data against a reference unit and subsequently applying weighting factors provided by the JRC. ^37^ ^,^ ^38^ This procedure ensures that different environmental indicators are converted into dimensionless scores and can be aggregated in a single score. As highlighted by Domingo-Morcillo et al., ^16^ this method is considered the most suitable since it provides a single score across 16 science-based impact categories, offering sufficient detail to identify critical points and propose corrective actions.

From the social dimension, the social impacts are assessed using the SHDB method, which is consistent with the Social Life Cycle Assessment proposed by UNEP. The SHDB method operationalizes the social impact through worker-hours, which serves as the fundamental activity variable for characterization. ^39^ The SHDB addresses the social impacts of multiple subcategories, which can be aggregated supporting comparative social assessment. SHDB is suitable for this study as it allows for a comprehensive screening of all integrated subcategories, providing a consistent global framework to identify social risks across the entire supply chain.

From the economic dimension, a Life Cycle Costing technique is applied. A conventional Life Cycle Costing (LCC) approach was adopted to prevent the double counting of economic impacts already accounted for within the environmental and social analysis. Furthermore, to ensure methodological coherence across the three pillars of sustainability, a consistent system boundary and scope were maintained. This involves the exclusion of depreciation costs, as existing capital expenditures represent a negligible value when normalized per declared unit. The economic analysis focuses exclusively on the operational costs associated with the FLWPR action and does not incorporate product sales prices. This strictly cost-based approach was chosen to avoid ‘positive/negative’ net results that could be influenced by market volatility. Accordingly, the economic results should be interpreted as an assessment of operational cost efficiency and not as a comprehensive economic sustainability assessment.

Regarding the databases and software used, the study relies on the Ecoinvent 3 – APOS database for evaluating environmental impacts and the SHDB v1.00 database for social impacts. All calculations are performed in SimaPro 9.1.0.8, using the Environmental Footprint 3.1 normalization and weighting set together with the SHDB methodology. The economic analysis is based on cost estimates derived from market price proxies.

This study applies the mass allocation criterion to allocate the environmental impacts. This physical partitioning method ensures that burdens are distributed proportionally to the mass of the final product and its associated co-products and loss and waste. Additionally, given the specific problem analyzed, this study corrects the impact results by the FLW ratio. In this way, the analysis explicitly considers the inefficiencies attributable to food loss and waste, thereby ensuring that the evaluation accurately reflects the environmental, social, and economic impacts per kilogram of fresh potato. This combined approach, mass allocation, FLW ratio correction, and conversion of elementary flows via EF 3.1 characterization factors and SHDB worker-hours, constitutes the complete methodological chain linking inventory inputs to the reported impact results.

## 4. Results and discussion

The comparison between the two scenarios was conducted both at the level of individual impact categories and at the aggregated level, using a single score that represents the sum of the weighted impact categories. The next subsections present the results by sustainability dimension.

### 4.1 Results of the accounting-based scenarios: without and with FLWPR action

**4.1.1 Environmental impact assessment**

**Figure 3.**
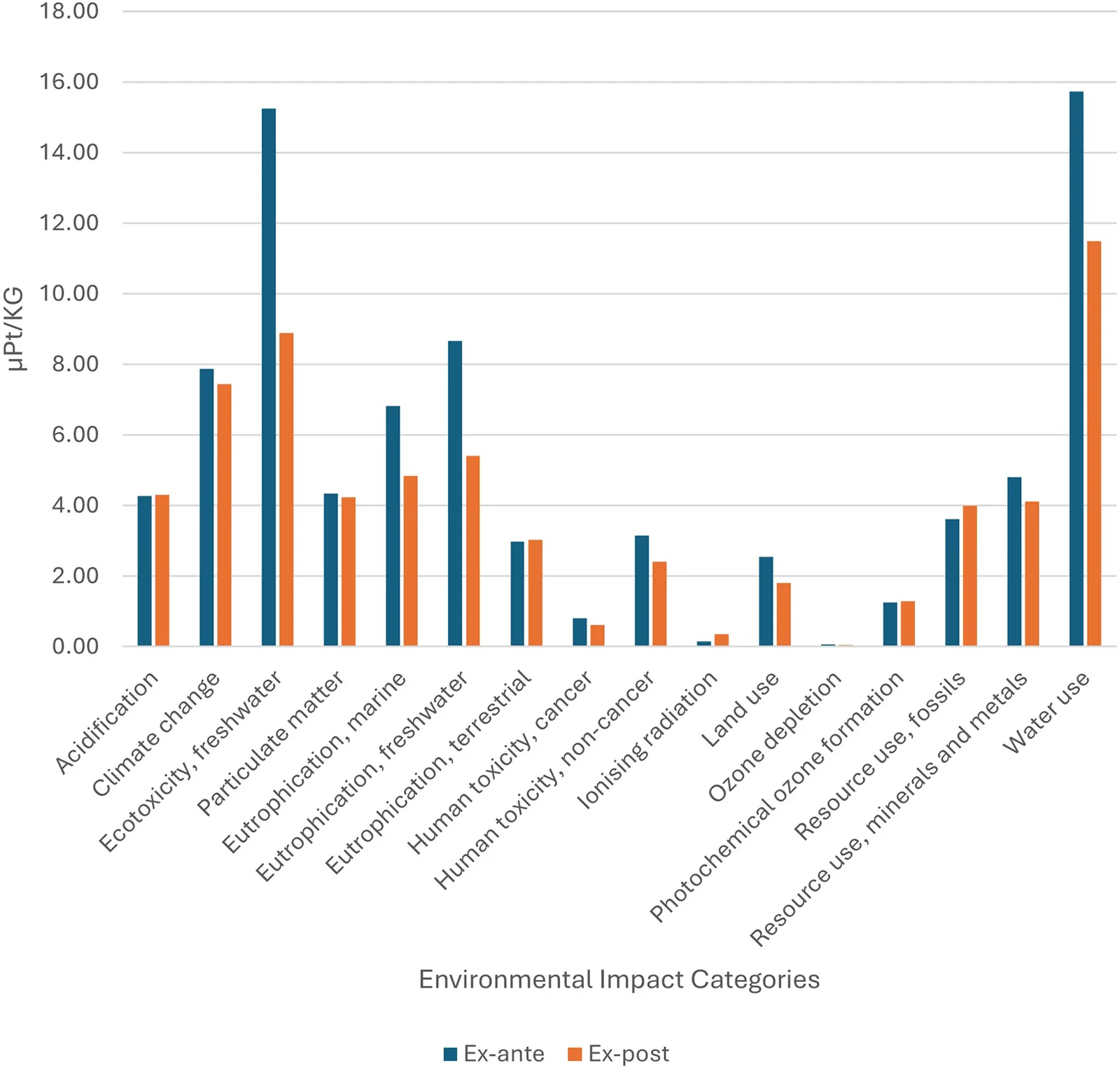
Environmental impact assessment. Comparative assessment of environmental impacts between the accounting-based scenario without and with FLWPR action, expressed as weighted results for each Environmental Footprint (EF) 3.1 impact category. Source: Own elaboration.

Figure 3 shows the environmental results of the accounting-based scenario without and with FLWPR action. The environmental sustainability assessment of the technological action implemented revealed significant environmental benefits across multiple impact categories. Specifically, the redistribution of 0.47 kg of potatoes yielded a total environmental reduction of 18.02 μPt/kg, with substantial decreases observed in ecotoxicity, water consumption, and eutrophication (see Table 3). The impact category with a higher contribution to the overall environmental impact in both scenarios is “Water Use”. The main source of this impact can be associated with the potato production stage. Accordingly, the improvement proposal should prioritize the development of new measures that specifically address this issue. For the current solution, the margin for further improvement is limited but remains feasible by directly reducing the amount of food wasted. The second most relevant impact category is “Climate Change,” followed by “Acidification” and “Particulate Matter.” Potential improvements in these categories could be achieved through efficiency studies focused on the transformation process, where technological innovations linked to reduced resource use are expected to play a pivotal role.

**Table 3.** Environmental impact reduction by kg of fresh potato.

Impact categories	Impact reduction (μPt/kg)
Acidification	-0.04
Climate change	0.42
Ecotoxicity, freshwater	6.36
Particulate matter	0.10
Eutrophication, marine	1.97
Eutrophication, freshwater	3.25
Eutrophication, terrestrial	-0.05
Human toxicity, cancer	0.20
Human toxicity, non-cancer	0.73
Ionising radiation	-0.20
Land use	0.74
Ozone depletion	0.02
Photochemical ozone formation	-0.02
Resource use, fossils	-0.37
Resource use, minerals and metals	0.69
Water use	4.24
**TOTAL**	**18.02**

Table shows the results of the environmental impact reductions achieved through the implemented food loss and waste prevention and reduction action. Note that a negative value indicates an increase in environmental impact and therefore does not represent a reduction.

Source: Own elaboration.

Table 3 presents the difference in weighted environmental impacts between the accounting-based scenario without and with FLWPR action. This comparison enables a clear quantification of the changes attributable to the intervention and supports informed decision-making based on the action’s sustainability performance. Overall, Table 3 shows a favorable reduction in total impact and across a large number of impact categories. However, five associated impact categories display an unfavorable evolution after the implementation of the FLWPR action. These categories can be identified as potential environmental risk points, as they indicate an increase in environmental burdens. This outcome is explained by the fact that the saved impacts due to the food waste reduction are not enough to offset the resource consumption and associated impacts of the technologies employed, both for early detection and for valorization processes. Nevertheless, in relative terms, the negative contribution of these 5 categories remains low compared with the overall reduction in impact.

**4.1.2 Social impact assessment**

The action also resulted in a reduction in social risk potential, quantified at 16.62 Pt/kg. In addition, it generated a well-balanced reduction across all five major social impact categories (see Table 4).

**Table 4.** Social impact reduction by kg. of fresh potato.

Impact categories and subcategories	Impact reduction (Pt/KG)
**Labor Rights & Decent Work**	**8.32**
Child Labor	1.06
Forced Labor	0.54
Excessive Working Time	0.53
Wage Assessment	2.66
Poverty	0.86
Migrant Labor	0.35
Collective Bargaining	1.86
Social Benefits	0.45
**Health & Safety**	**2.55**
Injuries and Fatalities	0.84
Toxics and Hazards	1.71
**Human Rights**	**1.94**
Indigenous Rights	0.45
Gender Equity	0.52
High Conflict	0.97
**Governance**	**2.07**
Legal System	0.92
Corruption	1.15
**Community Infrastructure**	**1.74**
Drinking Water	0.48
Improved Sanitation	0.57
Hospital Beds	0.69
**TOTAL**	**16.62**

Table shows the results of the social impact reductions achieved through the implemented food loss and waste prevention and reduction action.

Source: Own elaboration.

**Figure 4.**
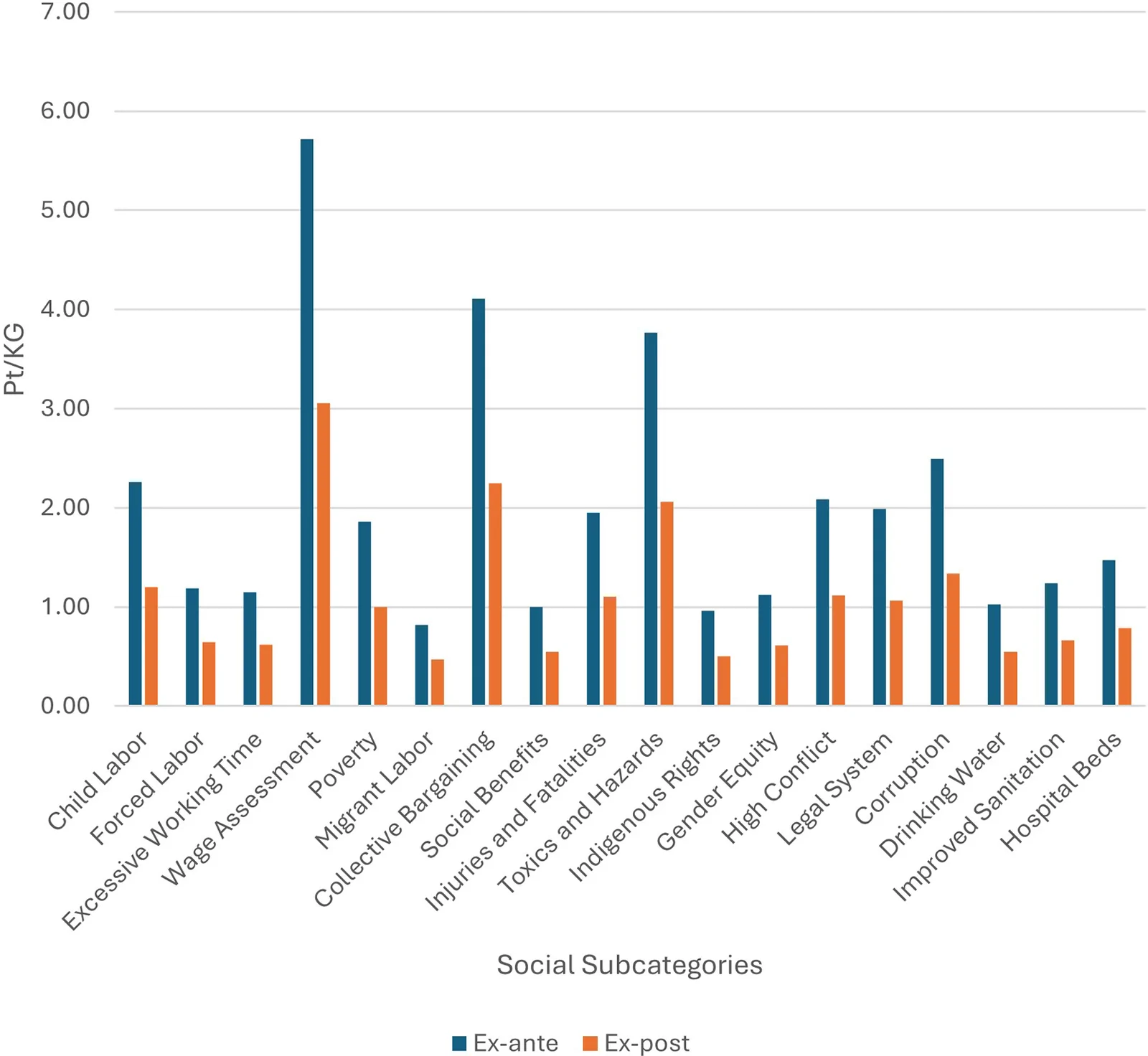
Social impact assessment. Comparative assessment of social impacts between the accounting-based scenario without and with FLWPR action, expressed as results for each SHDB impact subcategory. Source: Own elaboration.

Figure 4 displays the social results of both scenarios. The social subcategory with the highest contribution corresponds to “Wage Assessment”, with the main contribution originated from labor conditions in the potato production phase. Improvement efforts should therefore prioritize the development of new measures targeting this aspect. The second most relevant impact subcategory is Injuries and Fatalities. In third place, in terms of relative importance, is “Toxics and Hazards”, followed by “Collective Bargaining”. Across all social impact categories, the primary contribution stems from food-related processes. Consequently, improvement strategies should emphasize actions that directly address this source of impact. Regarding the current FLWPR action, the potential margin for improvement is limited but still possible, particularly by further reducing the amount of food loss and waste.

Table 4 presents the difference in social impacts between both scenarios. Table 4 presents the differences in social impacts between the two scenarios. The results indicate that all social impact categories have a reduction. The most substantial decrease is observed in “ *Labor Rights and Decent Work”*, which accounts for a total reduction of 8.32 Pt/kg. Within this category, the largest contributions to the improvement arise from wage assessment (2.66 Pt), collective bargaining (1.86 Pt), and child labor (1.06 Pt). These results highlight that the technological FLWPR action itself generates only a very limited social impact. Consequently, the reductions achieved in all social impact categories can be clearly attributed to the decrease in food loss and waste.

**4.1.3 Economic impact assessment**

From an economic perspective, the evaluation focuses exclusively on cost. Prior to the implementation of the action, the cost was 2.43 €/kg, largely due to the inefficiencies associated with food loss and waste, which reached rates of 49%. The technological FLWPR action reduced food loss and waste to an optimal level of approximately 2%. This improved resource use, enabled by early food waste and loss detection and the various waste valorization options, allowed for a more efficient allocation of resources. Even considering the additional resources required by the technological solution, the cost per kilogram decreased to 2.29 €/kg, representing a reduction due to the action of 0.14 €/kg. These figures reflect operational cost efficiency within the LCC scope defined in Section 3.3 and should not be interpreted as indicators of overall economic profitability or comprehensive economic sustainability. This improvement is explained by an increased efficiency in food handling, enhanced product preservation, and the optimization of human and energy resources. The reduction of waste at the distribution stage not only avoids direct losses but also enhances overall supply chain performance.

### 4.2 Results of the simulated scenarios according to the food waste prevention and reduction hierarchy

Table 5 shows the results of the sensitivity analysis conducted to assess how the sustainability outcome varies when different percentages are applied to each destination, where the use of the various resources is determined essentially by the share of the transformation process selected. Scenario E2 achieves the most favorable results in environmental terms by directing all loss to animal feed. Conversely, the environmental performance of Scenarios C, D, and E3 deteriorates compared to the accounting-based scenario, even without FLWPR action. This outcome highlights critical findings. On the one hand, the energy-intensive nature of starch processing creates an environmental burden that surpasses the benefits of recovery from food loss. On the other hand, strategies like 5th-range food production sit higher in the functional hierarchy, their high resource intensity makes them less efficient than animal feed in this specific context, justifying E2’s position as the optimal scenario in the environmental dimension. This is consistent with previous studies ^4^ ^,^ ^14^ ^,^ ^15^ that found that certain reduction strategies can be counterproductive. For instance, Muñoz-Torres et al., ^14^ demonstrated that processing surplus oranges into juice may result in higher environmental impacts than simple waste disposal due to the high energy and packaging demands of the industrial transformation.

**Table 5.** Sensitivity analysis: Sustainability impact reductions and food loss strategies.

Scenarios	Scenario A	Scenario B	Scenario C	Scenario D
5th range Animal feed Starch	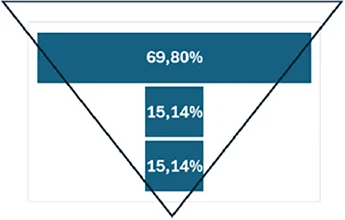	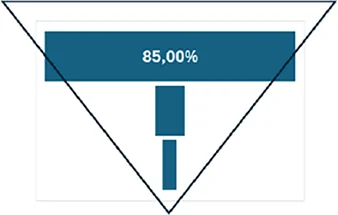	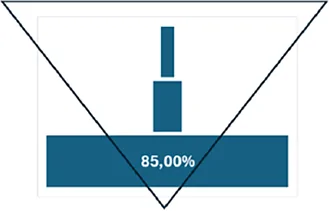	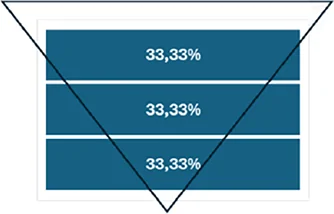
**Results**				
**Environmental**	18.02 μPt/kg	28.94 μPt/kg	−59.26 μPt/kg	−1.07 μPt/kg
**Social**	16.62 Pt/kg	16.97 Pt/kg	14.23 Pt/kg	16.00 Pt/kg
**Economic**	0.14 €/kg	0.14 €/kg	0.13 €/kg	0.14 €/kg

Scenarios		Scenario E1	Scenario E2	Scenario E3
5th range Animal feed Starch		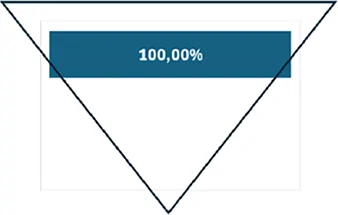	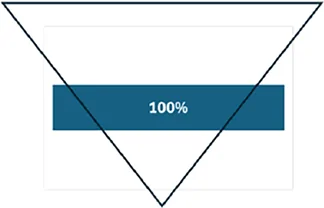	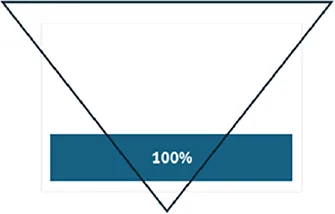
**Results**				
**Environmental**		33.93 μPt/kg	39.18 μPt/kg	−76.12 μPt/kg
**Social**		17.14 Pt/kg	17.13 Pt/kg	13.72 Pt/kg
**Economic**		0.13 €/kg	0.16 €/kg	0.12 €/kg

Regarding social impacts, all evaluated scenarios demonstrate a net reduction in social burden. This indicates that the social impacts avoided through food loss reduction effectively outweigh those generated by the new resources required for strategy implementation. Scenario E1 is the most favorable, closely followed by Scenario E2. This highlights that these scenarios either possess a lower resource intensity or are associated with more favorable social conditions in their respective supply chains.

In economic terms, all scenarios result in cost reductions. However, it is worth noting that animal feed shows the greatest cost reduction. This is once again explained by the efficiency of its production process, particularly due to the relatively low cost of consumption measured in euros.

By integrating the three perspectives, environmental, social, and economic, the analysis reveals that if the action owner has the necessary market access and infrastructure for animal feed production (Scenario E2), this emerges as the optimal pathway across all sustainability dimensions. However, less radical shifts, such as incrementally increasing the percentage of 5th-range food production over starch, also yield significant improvements compared to Scenario (A) which is the already implemented in a pilot phase.

These findings invite a critical discussion on two fronts. First, the transformation of losses into industrial products (recycling), such as starch, can incur a disproportionately high environmental cost. This suggests that certain ‘recycling’ tiers in the waste hierarchy may be critical tipping points where a dedicated environmental impact assessment is required to ensure that the recovery process does not cause more harm than the initial loss. Second, while the hierarchy typically prioritizes human consumption (5th-range foods), this study shows that animal feed can be a more sustainable alternative due to its low resource intensity. Finally, it is important to note that since the selling price of the end products was not included in this model, the action owner should integrate economic market data to complement these findings before final implementation.

To assess the robustness of the key finding that animal feed performs best across the assessed sustainability dimensions, a qualitative sensitivity assessment of key parameters was carried out. Detection rate is among the most sensitive parameters: a lower-than-modeled detection rate would reduce the avoidance of food losses, proportionally diminishing environmental and economic benefits across all scenarios. Electricity consumption is particularly influential for the starch and fifth-range scenarios; higher energy intensities would further widen the environmental performance gap relative to animal feed. Transformation yields determine the mass of co-products credited under mass allocation; lower yields reduce the allocated benefit and reinforce animal feed’s relative advantage. Finally, cost assumptions — including raw material prices and unit processing costs — are most sensitive in the starch and fifth-range pathways. Across all parameters examined, the direction of the sensitivity is consistent: variations in these assumptions do not alter the conclusion that animal feed represents the most robust option under the above-mentioned conditions.

## 5. Conclusions

This study presents a comprehensive sustainability assessment of a technological FLWPR action, which integrates multiple technologies to enhance food waste prevention and contribute to the development of a more sustainable food system. The technologies applied operate at different stages of maturity. On the one hand, pilot-phase technology was tested, designed to prevent waste through the implementation of an advanced detection system. This system employs appropriate sensors installed at the sorting stage of the packaging line to identify and remove potatoes with a high risk of generating losses due to defects, thereby preventing waste before it occurs. On the other hand, market-ready technologies were applied to valorize defective potatoes through the production of fifth-range products (ready-to-eat meals), animal feed, and industrial transformation. The sustainability assessment was conducted using a life cycle approach, integrating the three dimensions of sustainability, environmental, social, and economic, through methods widely recognized in academic research. The system boundaries for this multi-technological solution extended from production to consumption.

The first results based on the FLW rate demonstrated that the FLWPR solution effectively improved the identification of defective potatoes, significantly reducing waste generation and contributing to circular economy practices by redirecting defective potatoes to alternative uses. The sustainability results revealed positive impacts across all three dimensions. Environmentally, the intervention achieved notable reductions in ecotoxicity and water use, reflecting improved resource efficiency. Socially, the pilot generated reductions in modeled social risk potential across the subcategories of wage assessment, collective bargaining, and indicators related to forced and child labor, as assessed using the SHDB screening methodology. In terms of operational costs assessed under the LCC framework, the intervention reduced costs per kilogram of fresh potato, reflecting improved supply chain efficiency. A deeper analysis of the results highlights critical areas requiring continued attention, particularly certain environmental impact categories that showed increased risk.

Following the sustainability assessment of the action, a sensitivity analysis was conducted by varying the shares of food loss reduced to the different strategies positioned across the food waste hierarchy. The results show that the scenario in which all food loss and waste is diverted to animal feed achieves the best overall sustainability performance, particularly in environmental and economic terms, due to its low resource intensity and high-cost efficiency. In contrast, starch-based recovery and 5th-range food production perform worse environmentally than the baseline scenario, driven by the high energy and processing requirements of industrial transformation. These findings highlight that not all recovery pathways within the waste hierarchy are equally sustainable, and that certain industrial valorization processes may offset the environmental benefits of food loss reduction.

This article makes several contributions to the field of sustainability assessment of technological FLWPR actions. First, it provides a comprehensive sustainability evaluation that integrates environmental, social, and economic dimensions from a life cycle perspective, applying widely recognized scientific standards. This is particularly relevant in the technological assessment domain, where there is a notable lack of studies that include social impact evaluation. The methodology presented here offers a framework that can be applied not only to determine the economic feasibility of scaling up technologies but also to assess their contribution to sustainable development.

Second, the article establishes a direct link between the technical results obtained from life cycle impact measurement and the management system of the prevention and reduction action. This connection allows for the identification of hotspots, risks, and recommendations for improvement. From a practical standpoint, it is not only essential to decide whether technology should be adopted but also to understand how it can be continuously improved within a framework of ongoing optimization.

Third, the study evaluates a set of technologies at different levels of maturity, demonstrating how their combination can maximize the effectiveness of food waste prevention and reduction strategies. Emphasis is placed on the upper tiers of the food waste hierarchy, which remains underrepresented in scientific research, as most existing studies focus primarily on waste treatment rather than prevention. The positive results of this assessment suggest that this multi-technological approach may be of interest to other companies involved in potato distribution and sales. By adopting similar strategies, these companies could directly contribute to Sustainable Development Goal 12.3 as well as to other environmental, social, and economic goals associated with broader impact categories.

Fourth, an additional contribution of the sensitivity analysis is that it provides decision-makers with evidence that, beyond the waste hierarchy, each action should be evaluated in terms of its environmental, social, and economic performance. This is because the hierarchy of waste management options is not always fully aligned with the outcomes of a comprehensive sustainability assessment. As shown by the results, certain options that are theoretically higher in the hierarchy may not necessarily represent the most sustainable alternative. Therefore, a complementary decision-making approach that integrates life cycle-based sustainability criteria is recommended to support more robust and context-specific policy and management choices.

This paper acknowledges several limitations related to empirical analysis. One of the main limitations concerns the social datasets available in the specialized databases associated with the food analyzed, which are based on general averages without the possibility of selecting a dataset specific to the variety and region studied. Consequently, the reported social results reflect reductions in modeled social risk rather than measured improvements in actual labor conditions. As social databases continue to expand and improve, future evaluations will be able to achieve greater accuracy and site-specific relevance. Another limitation arises in the economic dimension, where the assessment currently considers only cost as an impact category. Future research should explore additional categories such as profit margins, eco-efficiency, and other indicators that can provide a more comprehensive understanding of economic performance. A further limitation is linked to the pilot phase of the early detection technology applied to potatoes. Previous studies suggest that the performance of such technologies may vary when scaled to market-level applications. If this methodology eventually reaches commercial maturity, repeating the sustainability assessment would allow for a comparative analysis to identify potential deviations across different stages of technological development. Another limitation arises in the economic dimension, where the LCC has been simplified to consider only specific operational cost categories. Fixed investment costs were excluded to maintain consistency with the LCA boundaries, as their impact on the functional unit was considered negligible. Furthermore, discount rates, time horizons and revenues were not included to provide a static, transparent assessment of operational costs, preventing markets volatility from biasing the technical performance results and ensuring methodological consistency with the non-discounted environmental LCA. Another relevant limitation of this study is that the technology was assessed on a pilot scale. While the results provide robust proof of concept, the life cycle impacts, particularly energy efficiency and economic costs, may vary when transitioning to full industrial-scale implementation. Future research should focus on validating these findings in large-scale operational environments to confirm the identified sustainability benefits.

Overall, this study confirms the value of integrating technological innovation with sustainability assessment frameworks to prevent food loss and waste. The findings support the scaling up of such solutions within the retail sector and provide actionable insights for stakeholders seeking to enhance sustainability performance across food supply chains.

## Ethical approval statement

This study involved human participants. Specifically, the owners of the action provided information regarding the technological FLWPR action within the ToNoWaste project through meetings and a questionnaire. The research was conducted in accordance with the ethical guidelines and regulations of the ToNoWaste Horizon Europe project and its coordinating institution. The study protocol was reviewed and approved by the Human Research Ethics Committee of Universitat Jaume I (Spain), with the ethical approval file number CEISH/56/2023.

## Consent to participate

The participants in this study are part of the consortium of the ToNoWaste Horizon Europe Project and they signed the Grant Agreement and the Consortium Agreement (25/05/2022), which included a written informed consent to participation in research.

## Declaration of generative AI and AI-assisted technologies in the manuscript preparation process

During the preparation of this work the authors used Microsoft Copilot tool (Microsoft, 2025) in order to improve readability and language. After using this tool, the authors reviewed and edited the content as needed and take full responsibility for the content of the published article.

## Data Availability

Repositori UJI, TONOWASTE (Towards a New Zero Food Waste Mindset Based on Holistic Assessment). https://repositori.uji.es/handle/10234/775844. ^36^ This project contains the following underlying data:
• Dataset of environmental, social and economic impact: a Multi-Technology Action for Food Loss and Waste Prevention. Data is available under the terms of the CC-BY 4.0 license.
