# Supplementary material for: Life cycle sustainability assessment of a multi-technology action for food loss and waste prevention
Source: Open Res Eur. 2026 Jul 9;6:44. Originally published 2026 Feb 13. [Version 3] doi: 10.12688/openreseurope.22916.3 (PMC13554740; doi:10.12688/openreseurope.22916.3)
Supplement: Peer review history

# Reviewer report: Reviewer response for version 3

**DOI:** 10.21956/openreseurope.26540.r77328

**Published:** 2026-08-14

**Authors:** Ana Marta-Costa

**Affiliations:** 1 University of Trás-os-Montes e Alto Douro (UTAD), Vila Real, Portugal

This paper addresses an important issue related to the prevention of food loss and waste, using a life cycle sustainability assessment of a multi-technology intervention. The topic is highly relevant, and the results appear promising and potentially impactful.

 The manuscript is generally well organized and clearly structured. However, it presents some weaknesses, particularly regarding the economic and social dimensions of sustainability. In addition, the methodological description lacks sufficient detail and should be further clarified, expanded, and strengthened.

 In the abstract, the statement that the study “demonstrates a substantial reduction in food loss and waste, a reduction in the impacts of the three pillars of sustainability, and a successful implementation of circular economy practices” requires clarification. Referring to a “reduction in the impacts” of the three pillars of sustainability may generate some confusion. While a reduction in environmental impacts is generally understood as positive, the interpretation is less straightforward when applied to the social and economic dimensions. This ambiguity remains in the results section. The relationship between the measured values and sustainability performance should be made clearer: does a higher value indicate better sustainability performance, or the opposite? Since interpretations may differ depending on the sustainability dimension considered, this should be explicitly explained in the manuscript.

 In the introduction and in Section 2, the authors refer to several limitations of life cycle assessment methodologies, particularly regarding the economic and social dimensions. However, it is not clear what methodological adaptations were made in the present study to address or mitigate these limitations. As currently presented, some of these limitations appear to have been reproduced rather than overcome. The choice of the methodology adopted should therefore be better justified, including a clearer explanation of why this approach was considered the most appropriate and why alternative methods were not selected. In addition, the literature review in Section 2 could be further strengthened in order to provide a more robust theoretical and methodological foundation for the selected approach within the broader literature on life cycle sustainability assessment.

 In the methodology section, the authors state that “fixed capital investments and infrastructure were excluded from the system boundary as they represent negligible impacts when normalized per declared unit.” This methodological choice may represent a weakness and should be better explained. Investment costs associated with the technology under study are relevant for decision-making and may affect the future economic and financial viability of the project, and therefore its economic sustainability. Moreover, excluding these costs may limit the possibility of comparing this technology with alternative technologies or even comparing different scenarios, since the outputs may differ substantially.

 Figure 1 presents quantities of food and losses associated with the “characterization of accounting-based scenarios.” However, it is not clear whether these quantities refer specifically to potatoes. How were these quantities obtained? What were the sources of information? Under what conditions was the data collected? The manuscript should provide more detail on the data collection process and on the sources used.

 The presentation of the seven scenarios used for the sensitivity analysis should also be further developed. In particular, the manuscript should clarify how the use of the technology differs across the different scenarios.

 The multi-technology intervention being tested is only briefly mentioned, and the manuscript does not provide sufficient information about what it consists of, how it operates, or how it is implemented in practice. A clearer and more detailed description of the intervention, including its components, operational mechanisms, and context of application, is necessary to allow readers to properly understand the intervention and assess its results.

 The manuscript should also explain how the different indicators presented in the results section were measured. What are the sources of the information used? How were the data collected and validated?

 Another relevant point concerns the replicability of the model. Can the proposed model be applied to other food products, or is it specific to the product analyzed in this study? This issue should be addressed more explicitly.

 In conclusion, while the paper addresses an important topic and presents relevant empirical results, substantial revisions are needed. Strengthening the theoretical foundation, providing clearer methodological details, and offering more contextual information about how, where, and from whom the information was collected would significantly improve the quality, credibility, and impact of the work.

 Therefore, I recommend acceptance of the manuscript subject to major revisions.

Is the study design appropriate and does the work have academic merit?

Partly

Is the work clearly and accurately presented and does it cite the current literature?

Partly

If applicable, is the statistical analysis and its interpretation appropriate?

No

Are all the source data underlying the results available to ensure full reproducibility?

No

Are the conclusions drawn adequately supported by the results?

Yes

Are sufficient details of methods and analysis provided to allow replication by others?

No

Reviewer Expertise:

Agribusiness and sustainability

I confirm that I have read this submission and believe that I have an appropriate level of expertise to confirm that it is of an acceptable scientific standard, however I have significant reservations, as outlined above.

# Reviewer report: Reviewer response for version 3

**DOI:** 10.21956/openreseurope.26540.r77326

**Published:** 2026-07-25

**Authors:** Konstantina Papadopoulou

**Affiliations:** 1 National Technical University of Athens, Zografou, Greece

The manuscript presents a Life Cycle Sustainability Assessment of a multi-technology intervention for food loss and waste prevention in the fresh potato supply chain, combining EF 3.1 for environmental impacts, SHDB for social impacts, and Life Cycle Costing for economic impacts. The revised version also includes a sensitivity analysis across seven scenarios reflecting different distributions of food loss across recovery pathways positioned at different levels of the food waste hierarchy.

 Two points remain that affect replicability and the strength of the conclusions:

**1. **Replication (methods and analysis). The sensitivity analysis added in Section 4.2 is a reasonable and proportionate response given the data available from a single month pilot, but it remains qualitative. Reporting direction of effect for detection rate, electricity use, transformation yields, and cost assumptions, without ranges, confidence intervals, or a formal variation exercise (e.g., ±X% on key parameters). A scientific reader attempting to replicate or stress test the analysis would need this quantitative detail to reproduce the robustness claim.

 --I recommend the authors either report a simple range based variation (e.g., ±10–20% on the most sensitive parameters) or explicitly state the qualitative nature of the exercise as a bounded limitation in the text. 

**2.** Conclusions supported by results. The headline conclusion, that directing all food loss to animal feed (Scenario E2) is the most sustainable pathway, is well supported environmentally, where the margin over other scenarios is substantial. However, on the social and economic dimensions, Scenario E1 (5th range foods) and E2 are close (17.14 vs. 17.13 Pt/kg socially; 0.13 vs. 0.16 €/kg economically), and the qualitative sensitivity analysis does not establish how robust this relative ordering is to parameter variation.

 --The conclusions section should note this more explicitly, distinguishing the strength of evidence for the environmental ranking from the comparatively narrower and less tested margins in the social and economic dimensions.

**A further minor point:** the underlying inventory is drawn from a single month of pilot data. This is disclosed as a limitation, but since the conclusions are generalized as a basis for recommending the approach to other retailers, an explicit caveat that seasonal variation in defect rates and detection performance has not been tested would keep this generalization appropriately bounded.

**Points that should be addressed to strengthen scientific soundness:**

- Report at least an indicative quantitative range for the sensitivity analysis, or explicitly bound the qualitative claim of "robustness" in the text.
- Add a sentence in Section 4.2 or the Conclusions distinguishing the confidence level of the environmental ranking from the closer social/economic margins between E1 and E2.
- Add a brief caveat on the untested seasonality of the single month inventory period where the findings are generalized to other retailers/contexts.

None of these require new data collection or a redesign of the study. Authors can be addressed through minor textual additions and a more cautious framing of the robustness claim.

Is the study design appropriate and does the work have academic merit?

Yes

Is the work clearly and accurately presented and does it cite the current literature?

Yes

If applicable, is the statistical analysis and its interpretation appropriate?

Not applicable

Are all the source data underlying the results available to ensure full reproducibility?

Yes

Are the conclusions drawn adequately supported by the results?

Partly

Are sufficient details of methods and analysis provided to allow replication by others?

Partly

Reviewer Expertise:

-Life Cycle Assessment (LCA) and Life Cycle Costing (LCC) of Waste Management Systems; -Circular Economy and Valorization of Agro-Industrial and Food Waste; -Municipal Solid Waste Characterization and Management; -Production of Biofuels (Hydrogen, Methane, Bioethanol) from Waste Biomass; -Industrial-Urban Symbiosis for Resource Recovery

I confirm that I have read this submission and believe that I have an appropriate level of expertise to confirm that it is of an acceptable scientific standard, however I have significant reservations, as outlined above.

# Response

**Published:** 2026-08-06

**Authors:** Idoya Ferrero-Ferrero

**Affiliations:** Sustainability of Organizations and CSR Management, University Jaume I Department of Finance and Accountancy, Castellón de la Plana, Valencian Community, Spain

Dear Reviewer, Thank you for your careful reading and constructive comments, which we address below.

 1. Sensitivity analysis (Section 4.2). The manuscript already presents a quantitative analysis across different scenarios for the destination of food waste, which is the key variable of this study. In addition, a sensitivity analysis was performed for other parameters, and the qualitative nature of this complementary exercise is explicitly stated in the text. We agree that this represents a limitation, and we consider that future studies could further explore variations in these parameters.

 2. Robustness of the conclusions. Given the qualitative nature of the sensitivity analysis, we cannot confirm the robustness of the relative ordering between Scenarios E1 and E2 on the social and economic dimensions. We encourage that this aspect be further investigated in future studies.

 3. Seasonality of the pilot data. We agree that this is a limitation inherent to the nature of the pilot from which the data were obtained, related to the untested seasonal variation in defect rates and detection performance. We thank the reviewer again for these valuable observations.

# Reviewer report: Reviewer response for version 3

**DOI:** 10.21956/openreseurope.26540.r77180

**Published:** 2026-07-15

**Authors:** Carlos Martin-Rios

**Affiliations:** 1 EHL Hospitality Business School, HES-SO University of Applied Sciences and Arts Western Switzerland, Delémont, Switzerland

The authors have carefully addressed all of my previous comments and concerns. The revisions have significantly strengthened the manuscript, and the responses to the reviewers are thorough and satisfactory. I have no further comments.

 I believe the paper is now ready for indexation. Congratulations to the authors on their work and on the successful revision of the manuscript.

Is the study design appropriate and does the work have academic merit?

Yes

Is the work clearly and accurately presented and does it cite the current literature?

Yes

If applicable, is the statistical analysis and its interpretation appropriate?

Not applicable

Are all the source data underlying the results available to ensure full reproducibility?

Yes

Are the conclusions drawn adequately supported by the results?

Partly

Are sufficient details of methods and analysis provided to allow replication by others?

Partly

Reviewer Expertise:

Sustainability innovation and management; food systems; food waste prevention and management

I confirm that I have read this submission and believe that I have an appropriate level of expertise to confirm that it is of an acceptable scientific standard.

# Reviewer report: Reviewer response for version 2

**DOI:** 10.21956/openreseurope.25892.r75364

**Published:** 2026-06-15

**Authors:** Carlos Martin-Rios

**Affiliations:** 1 EHL Hospitality Business School, HES-SO University of Applied Sciences and Arts Western Switzerland, Delémont, Switzerland

The article presents a LCSA of a multi-technology FL&W prevention and reduction action in the fresh potato supply chain. The study assesses environmental impacts using EF 3.1, social impacts through an SHDB-based approach, and economic impacts through life cycle costing.

 The topic is timely and relevant. The article makes a useful contribution by focusing on upstream food waste prevention technologies, which remain less frequently assessed than downstream waste treatment options. The revised version is also stronger because it examines several scenarios across the food waste hierarchy and shows that options ranked higher in the hierarchy are not always the most sustainable once environmental, social and economic criteria are considered together. This is a valuable insight for both researchers and practitioners. A few points could be strengthened to improve reproducibility and the robustness of the conclusions.

 First, the methods section should provide a clearer link between the inventory data and the reported impact results. The authors state that primary data were collected from the retailer and complemented with Ecoinvent and SHDB data, and that raw data are available. This is helpful. However, readers would benefit from a more explicit explanation of how the main inventory flows, database processes, allocation choices, and FLW correction were translated into the final environmental, social and economic results. A short supplementary table mapping the main inputs to the corresponding datasets and impact calculations would substantially improve replicability.

 Second, the scenario analysis is useful, but the article should more clearly distinguish scenario analysis from uncertainty analysis. The added scenarios help compare alternative valorization pathways, but they do not fully test uncertainty in key parameters. I recommend adding a concise sensitivity analysis on the most influential assumptions (i.e., detection rate, electricity use, labor requirements, transformation yields, and cost assumptions). This does not need to be extensive, but it would strengthen confidence in the conclusion that animal feed performs best across the assessed dimensions.

 Third, the economic analysis should be framed more carefully. The authors explain that they use a conventional cost-based LCC and exclude revenues, fixed investments, discount rates, and time horizons. This is acceptable if the aim is to assess operational costs, but the conclusions should avoid implying a full economic sustainability assessment.

 Fourth, the social assessment should be interpreted with caution. The SHDB approach is appropriate for a screening-level social assessment, but it relies on generic sectoral and country-level risk data. Therefore, the reported reductions should be described as reductions in modeled social risk or social impact potential, rather than direct evidence of improved labor conditions. Clarifying this point would make the social conclusions more accurate and credible.

Is the study design appropriate and does the work have academic merit?

Yes

Is the work clearly and accurately presented and does it cite the current literature?

Yes

If applicable, is the statistical analysis and its interpretation appropriate?

Not applicable

Are all the source data underlying the results available to ensure full reproducibility?

Yes

Are the conclusions drawn adequately supported by the results?

Partly

Are sufficient details of methods and analysis provided to allow replication by others?

Partly

Reviewer Expertise:

Sustainability innovation and management; food systems; food waste prevention and management

I confirm that I have read this submission and believe that I have an appropriate level of expertise to confirm that it is of an acceptable scientific standard, however I have significant reservations, as outlined above.

# Response

**Published:** 2026-07-03

**Authors:** Idoya Ferrero-Ferrero

**Affiliations:** Sustainability of Organizations and CSR Management, University Jaume I Department of Finance and Accountancy, Castellón de la Plana, Valencian Community, Spain

First, we would like to thank you for all the comments made by the review team, which helped us improve the paper. We have incorporated them into the new version of the document that has been uploaded, and changes have been highlighted. The next paragraphs collect the specific answers to the reviewer’ comments to clarify the text.

**ANSWERS TO THE COMMENTS FROM REVIEWER**

 Thank you for your comments. They are both pertinent and relevant and result from a careful paper analysis. They have helped us improve the analysis significantly.  We have regarded each comment in particular, trying to shed light and elaborate on points that needed to be upheld and expanded per your suggestions. For the sake of a clear organization, we have answered each of your suggestions separately. We agree that a number of key issues have improved since the revision.

**Comments** *1.- “First, the methods section should provide a clearer link between the inventory data and the reported impact results. The authors state that primary data were collected from the retailer and complemented with Ecoinvent and SHDB data, and that raw data are available. This is helpful. However, readers would benefit from a more explicit explanation of how the main inventory flows, database processes, allocation choices, and FLW correction were translated into the final environmental, social and economic results. A short supplementary table mapping the main inputs to the corresponding datasets and impact calculations would substantially improve replicability.”*  

 We thank the reviewer for this suggestion. We have clarified this point in Section 3.2, where we now explain more explicitly how the collected data was processed with the help of the specialized databases and the SimaPro software to build the life cycle inventory.  

*2.- “Second, the scenario analysis is useful, but the article should more clearly distinguish scenario analysis from uncertainty analysis. The added scenarios help compare alternative valorization pathways, but they do not fully test uncertainty in key parameters. I recommend adding a concise sensitivity analysis on the most influential assumptions (i.e., detection rate, electricity use, labor requirements, transformation yields, and cost assumptions). This does not need to be extensive, but it would strengthen confidence in the conclusion that animal feed performs best across the assessed dimensions.”*

 We thank the reviewer for this comment. We have addressed this point in Section 4.2, where we now clarify the sensitivity analysis carried out, examining how the sustainability outcome varies when different percentages are applied to each destination. In addition, complementing the key finding that animal feed performs best across the assessed sustainability dimensions, a qualitative sensitivity assessment of the key parameters was carried out.

*3.-“Third, the economic analysis should be framed more carefully. The authors explain that they use a conventional cost-based LCC and exclude revenues, fixed investments, discount rates, and time horizons. This is acceptable if the aim is to assess operational costs, but the conclusions should avoid implying a full economic sustainability assessment.”*  

 We thank the reviewer for this observation. The reviewer is right, and we have clarified this in the LCC analysis. Our analysis focuses on operational costs through a conventional cost-based LCC, and therefore does not constitute a full economic sustainability assessment.**

*4.- “Fourth, the social assessment should be interpreted with caution. The SHDB approach is appropriate for a screening-level social assessment, but it relies on generic sectoral and country-level risk data. Therefore, the reported reductions should be described as reductions in modeled social risk or social impact potential, rather than direct evidence of improved labor conditions. Clarifying this point would make the social conclusions more accurate and credible.”*  

 The reviewer is right, and we have revised the text accordingly, so that the reported reductions are now described as reductions in modeled social risk, rather than as direct evidence of improved labor conditions, in line with the screening-level nature of the SHDB approach.     Thank you again for your comments. We hope that the revised manuscript has improved enough to address your suggestions.

# Reviewer report: Reviewer response for version 1

**DOI:** 10.21956/openreseurope.24778.r71908

**Published:** 2026-04-13

**Authors:** Majid Bahramian

**Affiliations:** 1 Technological University Dublin, Dublin, Ireland

**1. Summary of the Article**

 The manuscript presents a Life Cycle Sustainability Assessment (LCSA) of a multi-technology intervention aimed at reducing food loss and waste along the supply chain. The study compares baseline and intervention scenarios, quantifying environmental impacts (LCA) and economic performance (LCC). The work attempts to evaluate the effectiveness of technological and management strategies in reducing food waste across stages such as production, distribution, retail, and consumption.

 The topic is relevant and timely, particularly in the context of circular economy strategies and food system sustainability. However, the manuscript exhibits **significant methodological limitations**, particularly in the LCA framework, system definition, uncertainty treatment, and economic analysis, which currently limit its scientific robustness.

**2. Major Comments (Must Be Addressed for Scientific Soundness)**

**2.1 LCA Framework and Transparency**

 The manuscript lacks fundamental components required for a scientifically sound LCA:

- The **reference flow and environmental matrix (technosphere matrix)** are not defined.
- The **link between inventory flows and impact categories (characterization factors)** is not explained.
- The **functional unit is not clearly operationalized within the modeling framework**.

These elements are essential for reproducibility and must be explicitly presented.

**2.2 System Boundary Definition**

 The system boundary is:

- Not clearly defined
- Overly simplified
- Lacking justification

There is no clear indication whether the system is:

- cradle-to-gate
- cradle-to-grave
- cradle-to-cradle

Additionally:

- **Water balances are not included**, which is a critical omission for food systems.

The authors must:

- Clearly define system boundaries
- Justify exclusions
- Ensure consistency with the functional unit

**2.3 Allocation Procedures**

 The manuscript does not clearly state:

- Whether allocation is applied
- Which allocation method is used (mass, economic, system expansion)
- Why the chosen method is appropriate

Given the multi-output nature of food systems, **allocation is unavoidable** and must be transparently addressed.

**2.4 Misinterpretation of Ex-ante LCA (Critical Issue)**

 The manuscript incorrectly classifies the analysis as **ex-ante LCA**.

- Ex-ante LCA refers to **future-oriented modeling of emerging technologies under uncertainty**
- Figure 1 presents **fixed, deterministic mass flows**, with no indication of:
  - scenario assumptions
  - adoption rates
  - uncertainty ranges

This is **not consistent with ex-ante LCA**, but rather resembles a **deterministic or accounting-based scenario**.

**This must be corrected**, either by:

- redefining the study as scenario-based LCA, or
- incorporating proper prospective modeling elements

**2.5 Lack of Uncertainty Assessment (Critical)**

 The manuscript does not include any meaningful uncertainty analysis.

 Given that LCA results are highly sensitive to assumptions, this is a **major methodological weakness**.

 The authors should include:

- **Sensitivity analysis** (minimum requirement)
  - e.g., ±10–30% variation in key parameters
  - alternative allocation methods
- **Scenario analysis**
  - baseline vs intervention pathways
  - different technological configurations
- **Monte Carlo simulation (recommended)**
  - assign probability distributions
  - report confidence intervals and overlaps
- **Data quality assessment (e.g., pedigree matrix)**

Without uncertainty assessment, results cannot be considered robust or decision-relevant.

**2.6 Justification of Case Study (Potato)**

 The choice of **potato** as the sole case study is not justified.

- Potato has relatively **low environmental impact compared to animal-based products**
- The rationale for its selection is unclear

The authors should:

- Justify the selection
- Or discuss limitations in generalizing results

**2.7 Terminology Issues**

 The manuscript uses inconsistent terminology:

- “Sustainability LCA”
- “Environmental LCA”

LCA is already an environmental assessment tool.

 If LCSA is intended, the integration of environmental, economic, and social pillars must be clearly demonstrated.

**2.8 Waste Management Pathways**

 The fate of waste streams is not clearly described:

- What happens to diverted food waste?
- How are downstream processes modeled?

This is essential for both LCA and LCC consistency.

**2.9 Life Cycle Costing (LCC) Methodology**

 The LCC component lacks transparency and rigor:

 Missing elements:

- Cost categories (CAPEX, OPEX, end-of-life)
- System boundaries (alignment with LCA unclear)
- Discount rate and time horizon
- Treatment of revenues / avoided costs

Additionally, no sensitivity analysis is performed on:

- energy prices
- discount rate
- technology costs

This significantly limits the reliability of the economic conclusions.

Is the study design appropriate and does the work have academic merit?

Partly

Is the work clearly and accurately presented and does it cite the current literature?

No

If applicable, is the statistical analysis and its interpretation appropriate?

No

Are all the source data underlying the results available to ensure full reproducibility?

No

Are the conclusions drawn adequately supported by the results?

No

Are sufficient details of methods and analysis provided to allow replication by others?

No

Reviewer Expertise:

‪Life Cycle Assessment‬ - ‪water and wastewater treatment‬

I confirm that I have read this submission and believe that I have an appropriate level of expertise to state that I do not consider it to be of an acceptable scientific standard, for reasons outlined above.

# Response

**Published:** 2026-05-06

**Authors:** Idoya Ferrero-Ferrero

**Affiliations:** Sustainability of Organizations and CSR Management, University Jaume I Department of Finance and Accountancy, Castellón de la Plana, Valencian Community, Spain

Thank you for your comments. They are both pertinent and relevant and result from a careful paper analysis. They have helped us improve the analysis significantly. We have regarded each comment in particular, trying to shed light and elaborate on points that needed to be upheld and expanded per your suggestions. For the sake of a clear organization, we have answered each of your suggestions separately. We agree that a number of key issues have improved since the revision.

***1. LCA Framework and Transparency***

*The manuscript lacks fundamental components required for a scientifically sound LCA:*

*•    The reference flow and environmental matrix (technosphere matrix) are not defined.*

*•    The link between inventory flows and impact categories (characterization factors) is not explained.*

*•    The functional unit is not clearly operationalized within the modeling framework.*

*These elements are essential for reproducibility and must be explicitly presented.*

 In response to the comments, we have clarified the link between inventory flows and impact categories in the life cycle impact assessment and we have clarified the declared unit used in the case study.  

***2 System Boundary Definition***

*The system boundary is:*

*•    Not clearly defined*

*•    Overly simplified*

*•    Lacking justification*

*There is no clear indication whether the system is:*

*•    cradle-to-gate*

*•    cradle-to-grave*

*•    cradle-to-cradle* *Additionally:*

*•    Water balances are not included, which is a critical omission for food systems.* *The authors must:*

*•    Clearly define system boundaries*

*•    Justify exclusions*

*•    Ensure consistency with the functional unit* We appreciate the reviewer’s feedback regarding the clarity and justification of the system boundary. We have revised section “3.1 Goal and scope” to explicitly define the scope, system boundaries and justify the exclusions. We appreciate the reviewer’s observation. While a physical mass balance for water has not explicitly detailed in the inventory section, the impacts related to water consumption were fully accounted for within the Water Use category, as defined by the EF 3.1 (Environmental Footprint) methodology. This characterization includes the water scarcity and consumption impacts associated with all upstream and core processes, including raw material production and the electricity mix.  

***3 Allocation Procedures***

*The manuscript does not clearly state:*

*•    Whether allocation is applied*

*•    Which allocation method is used (mass, economic, system expansion)*

*•    Why the chosen method is appropriate*

*Given the multi-output nature of food systems, allocation is unavoidable and must be transparently addressed.*

 We have clarified in section “3.3 Life cycle impact assessment” that we have applied a physical allocation method based on mass to distribute the environmental burdens across the system. Given the nature of the maincrop potato processing line, mass allocation was considered the most robust and transparent approach to reflect the physical flows of the multi-output process.

***4 Misinterpretation of Ex-ante LCA (Critical Issue)***

*The manuscript incorrectly classifies the analysis as ex-ante LCA.*

*•    Ex-ante LCA refers to future-oriented modeling of emerging technologies under uncertainty*

*•    Figure 1 presents fixed, deterministic mass flows, with no indication of:*

*o    scenario assumptions*

*o    adoption rates*

*o    uncertainty ranges*

*This is not consistent with ex-ante LCA, but rather resembles a deterministic or accounting-based scenario.*

*This must be corrected, either by:*

*•    redefining the study as scenario-based LCA, or*

*•    incorporating proper prospective modeling elements*

 We acknowledge the reviewer's clarification regarding the definition of ex-ante and ex-post LCA. Consequently, the study has been redefined as a scenario-based LCA using an accounting-based approach. We have renamed the former 'ex-ante' and 'ex-post' stages as “accounting-based scenario without FLWPR action” and “accounting-based scenario with FLWPR action”, respectively. Furthermore, as suggested, we have incorporated a scenario-based analysis to address different FLWPR strategies, moving beyond the initial deterministic model.

**5 Lack of Uncertainty Assessment (Critical)**

*The manuscript does not include any meaningful uncertainty analysis.*

*Given that LCA results are highly sensitive to assumptions, this is a major methodological weakness.*

*The authors should include:*

*•    Sensitivity analysis (minimum requirement)*

*o    e.g., ±10–30% variation in key parameters*

*o    alternative allocation methods*

*•    Scenario analysis*

*o    baseline vs intervention pathways*

*o    different technological configurations*

*•    Monte Carlo simulation (recommended)*

*o    assign probability distributions*

*o    report confidence intervals and overlaps*

*•    Data quality assessment (e.g., pedigree matrix)*

 To address these concerns, we have strengthened the manuscript by incorporating a comprehensive Sensitivity and Scenario Analysis, which effectively characterizes the uncertainty inherent in our assumptions. Regarding the sensitivity analysis, the study analyses the sensitivity of sustainability impacts to different allocation strategies and technological pathways (e.g., starch production vs. animal feed). This allowed us to identify critical "tipping points," such as the high energy intensity of starch processing, which can offset the benefits of loss reduction. For that we have defined and evaluated seven distinct scenarios (A–E) that compare the current industrial baseline (Scenario A) against various intervention pathways aligned with the waste hierarchy (Scenarios B–D) and extreme technological configurations (Scenarios E1–E3).

***6 Justification of Case Study (Potato)***

*The choice of potato as the sole case study is not justified.*

*•    Potato has relatively low environmental impact compared to animal-based products*

*•    The rationale for its selection is unclear*

*The authors should:*

*•    Justify the selection*

*•    Or discuss limitations in generalizing results*

 We have updated the text to justify the selection of the potato by highlighting that, according to the JRC, it accounts for 10% of all EU food waste, a figure in mass equivalent to the entire meat category. Furthermore, we have emphasized its status as the EU's primary non-cereal crop, making it a critical model for analyzing high-volume losses across the entire supply chain.

***7 Terminology Issues***

*The manuscript uses inconsistent terminology:*

*•    “Sustainability LCA”*

*•    “Environmental LCA”*

*LCA is already an environmental assessment tool.*

*If LCSA is intended, the integration of environmental, economic, and social pillars must be clearly demonstrated.*

 We have carefully revised the manuscript to address the reviewer’s comments. Specifically, we have replaced 'Environmental LCA' with 'LCA' throughout the text where appropriate to ensure terminology consistency. Regarding the integration of the three sustainability dimensions, we have now jointly analyzed them within the newly added section focused on the proposed scenarios.

***8 Waste Management Pathways***

*The fate of waste streams is not clearly described:*

*•    What happens to diverted food waste?*

*•    How are downstream processes modeled?*

*This is essential for both LCA and LCC consistency.* The fate of waste streams has been clarified. As highlighted by the action owner, the food loss and waste generated is treated through composting processes.

***9 Life Cycle Costing (LCC) Methodology***

*The LCC component lacks transparency and rigor:*

*Missing elements:*

*•    Cost categories (CAPEX, OPEX, end-of-life)*

*•    System boundaries (alignment with LCA unclear)*

*•    Discount rate and time horizon*

*•    Treatment of revenues / avoided costs*

*Additionally, no sensitivity analysis is performed on:*

*•    energy prices*

*•    discount rate*

*•    technology costs*

*This significantly limits the reliability of the economic conclusions.*

 We have enhanced LCC transparency by explicitly defining the system boundaries to ensure full alignment with the LCA. Any remaining aspect regarding the LCC applied has been formally addressed in the updated limitations in Conclusion section. Thank you again for your comments. We hope that the revised manuscript has improved enough to address your suggestions.

# Reviewer report: Reviewer response for version 1

**DOI:** 10.21956/openreseurope.24778.r70631

**Published:** 2026-03-13

**Authors:** Omar Ouro-Salim

**Affiliations:** 1 Universidade Nove de Julho, São Paulo, State of São Paulo, Brazil

The introduction of the article could be more concise and direct. At present, some paragraphs are excessively long and there is repetition of ideas related to the Sustainable Development Goals (SDGs) and food waste. It is recommended to slightly reduce the length of the introduction and emphasize more clearly the existing gap in the literature as well as the specific contribution of the article.

 The justification of the study could also be reinforced. Although the authors state that there are few studies on technologies for preventing food waste, it would be important to support this gap with more recent references. It is suggested to include additional studies on technologies aimed at food waste prevention and on the application of Life Cycle Assessment (LCA) in the food supply chain.

 Regarding the methodology, although it is adequate, some parts could be explained more clearly. In particular, it would be useful to detail the criteria for selecting social indicators, the method of aggregating social impacts, and the justification for using the SHDB method. This deeper explanation would help readers who are not familiar with Social LCA.

 In the Life Cycle Inventory section, the authors mention that the data were obtained in meetings with the retailer participating in the project. However, it would be important to clarify how these data were verified and whether complementary secondary data were used to strengthen the analysis.

 The discussion of the results presents relevant information but could be more in-depth. A comparison with other studies in the literature on food waste prevention and on screening or early defect detection technologies would enrich the analysis and provide greater context to the findings.

 Some figures also deserve more detailed explanations in the text. For example, Figure 1, which shows the food flow in the ex-ante and ex-post scenarios, could have its interpretation expanded to facilitate readers’ understanding.

 As for the language, although the English is clear, it is recommended to review some long sentences and simplify certain constructions. This would contribute to improving the fluency and readability of the text.

 Finally, the limitations section could be expanded. The authors mention restrictions related to the use of social data based on averages and to the economic analysis limited to cost. It is suggested to briefly add limitations related to the pilot scale of the technology, which would provide greater transparency to the study.

Is the study design appropriate and does the work have academic merit?

Partly

Is the work clearly and accurately presented and does it cite the current literature?

Partly

If applicable, is the statistical analysis and its interpretation appropriate?

Not applicable

Are all the source data underlying the results available to ensure full reproducibility?

Partly

Are the conclusions drawn adequately supported by the results?

Partly

Are sufficient details of methods and analysis provided to allow replication by others?

Partly

Reviewer Expertise:

Circular Economy.

I confirm that I have read this submission and believe that I have an appropriate level of expertise to confirm that it is of an acceptable scientific standard, however I have significant reservations, as outlined above.

# Response

**Published:** 2026-05-06

**Authors:** Idoya Ferrero-Ferrero

**Affiliations:** Sustainability of Organizations and CSR Management, University Jaume I Department of Finance and Accountancy, Castellón de la Plana, Valencian Community, Spain

Thank you for your comments. They are both pertinent and relevant and result from a careful paper analysis. They have helped us improve the analysis significantly. We have regarded each comment in particular, trying to shed light and elaborate on points that needed to be upheld and expanded per your suggestions. For the sake of a clear organization, we have answered each of your suggestions separately. We agree that a number of key issues have improved since the revision.

 1. The introduction of the article could be more concise and direct. At present, some paragraphs are excessively long and there is repetition of ideas related to the Sustainable Development Goals (SDGs) and food waste. It is recommended to slightly reduce the length of the introduction and emphasize more clearly the existing gap in the literature as well as the specific contribution of the article. We have improved the introduction by removing the initial paragraphs of SDGs and we have reinforced the research gap with additional references, focusing on the lack of multidimensional studies that validate the food waste hierarchy beyond the environmental dimension.

 2. The justification of the study could also be reinforced. Although the authors state that there are few studies on technologies for preventing food waste, it would be important to support this gap with more recent references. It is suggested to include additional studies on technologies aimed at food waste prevention and on the application of Life Cycle Assessment (LCA) in the food supply chain. We have added new references to substantiate this statement, specifically highlighting the existing gap between waste valorization and prevention technologies

 3. Regarding the methodology, although it is adequate, some parts could be explained more clearly. In particular, it would be useful to detail the criteria for selecting social indicators, the method of aggregating social impacts, and the justification for using the SHDB method. This deeper explanation would help readers who are not familiar with Social LCA. Thank you for your comment. We have expanded the “3.3 life cycle impact assessment” section to clarify the social assessment framework.

 4. In the Life Cycle Inventory section, the authors mention that the data were obtained in meetings with the retailer participating in the project. However, it would be important to clarify how these data were verified and whether complementary secondary data were used to strengthen the analysis. We have clarified the data verification process in the LCI section. Primary data provided by the retailer were supervised and validated by an agri-food sector expert to ensure technical consistency.

 5. The discussion of the results presents relevant information but could be more in-depth. A comparison with other studies in the literature on food waste prevention and on screening or early defect detection technologies would enrich the analysis and provide greater context to the findings. Thank you for your suggestion. We have enriched the discussion with an extra sensitive analysis and we have included references.

 6. Some figures also deserve more detailed explanations in the text. For example, Figure 1, which shows the food flow in the ex-ante and ex-post scenarios, could have its interpretation expanded to facilitate readers’ understanding. We have explained better Figure 1.

 7. As for the language, although the English is clear, it is recommended to review some long sentences and simplify certain constructions. This would contribute to improving the fluency and readability of the text. We have carefully revised the manuscript to improve clarity and readability.

 8. Finally, the limitations section could be expanded. The authors mention restrictions related to the use of social data based on averages and to the economic analysis limited to cost. It is suggested to briefly add limitations related to the pilot scale of the technology, which would provide greater transparency to the study.

 Thank you for the suggestion. It has been included in the limitations of the study in the Conclusion section. Thank you again for your comments. We hope that the revised manuscript has improved enough to address your suggestions.
